# A novel engineered strain of *Methylorubrum extorquens* for methylotrophic production of glycolic acid

**DOI:** 10.1186/s12934-024-02583-y

**Published:** 2024-12-23

**Authors:** Katharina Dietz, Carina Sagstetter, Melanie Speck, Arne Roth, Steffen Klamt, Jonathan Thomas Fabarius

**Affiliations:** 1 Fraunhofer Institute for Interfacial Engineering and Biotechnology, Straubing Branch BioCat, Schulgasse 11a, Straubing, Germany; 2 Max Planck Institute for Dynamics of Complex Technical Systems, Sandtorstr. 1, Magdeburg, Germany

**Keywords:** Bioeconomy, C_1_ fermentation, Methylotrophy, Synthetic methylotrophy, Systems biotechnology, Systems metabolic engineering, COBRA modeling, Metabolic core model, Bioprocess development, Bioengineering, Glycolate, Glycolic acid, Lactate, Lactic acid, Glyoxylate reductase, Serine cycle

## Abstract

The conversion of CO_2_ into methanol depicts one of the most promising emerging renewable routes for the chemical and biotech industry. Under this regard, native methylotrophs have a large potential for converting methanol into value-added products but require targeted engineering approaches to enhance their performances and to widen their product spectrum. Here we use a systems-based approach to analyze and engineer *M. extorquens* TK 0001 for production of glycolic acid. Application of constraint-based metabolic modeling reveals the great potential of *M. extorquens* for that purpose, which is not yet described in literature. In particular, a superior theoretical product yield of 1.0 C-mol_Glycolic acid_ C-mol_Methanol_^−1^ is predicted by our model, surpassing theoretical yields of sugar fermentation. Following this approach, we show here that strain engineering is viable and present 1st generation strains producing glycolic acid via a heterologous NADPH-dependent glyoxylate reductase. It was found that lactic acid is a surprising by-product of glycolic acid formation in *M. extorquens*, most likely due to a surplus of available NADH upon glycolic acid synthesis. Finally, the best performing strain was tested in a fed-batch fermentation producing a mixture of up to total 1.2 g L^−1^ glycolic acid and lactic acid. Several key performance indicators of our glycolic acid producer strain are superior to state-of-the-art synthetic methylotrophs. The presented results open the door for further strain engineering of the native methylotroph *M. extorquens* and pave the way to produce two promising biopolymer building blocks from green methanol, i.e., glycolic acid and lactic acid.

**Supplementary Information:**

The online version contains supplementary material available at 10.1186/s12934-024-02583-y.

## Introduction

The anthropogenic Global Warming is one of the greatest challenges of our time that must be addressed with major, multi-faceted, and coordinated effort. One promising path to a green chemical industry is to replace petrochemical routes with biotechnological processes applying microbial cell factories for the conversion of renewable resources [1–5]. The current development is directed by an increasing demand for sustainably synthesized products ranging from fine and bulk chemicals to synthetic fuels. This pull for sustainable production technologies is not only related to environmental issues of fossil-based processes (e.g. CO_2_ emissions), but also to the use of toxic solvents, high temperatures and energy consumption in conventional process routes [6].

A major advantage of microbial cell factories is that a vast variety of products can be derived by rewiring the metabolism and implementing non-native heterologous pathways through metabolic engineering [7]. Many metabolic engineering studies on various host microbes and products have been conducted successfully in recent decades [2, 8–10]. It is predicted that increasing costs for fossil feedstock and avoidance of associated CO_2_ emissions will be driving forces for microbial strain engineering that should not be underestimated in future scenarios [6].

In general, glucose-rich feedstreams are applied in fermentation processes. Glucose is sourced from agriculturally produced starch and sugar crops and, to a certain extent, also from wastes and residues, e.g. from cellulosic biomass [6]. However, the agricultural production of feedstock for fermentation requires arable land and thus competes with food production [11]. To overcome this issue, non-food feedstock like CO_2_/CO/H_2_-rich flue gas streams (i.e. synthesis gas) for gas fermentation processes has gained attention [12–16]. But currently available sources of synthesis gas are mainly associated with utilization of fossil resources [17]. In this context, a forgotten approach, essentially independent of fossil or agriculturally produced raw materials, has undergone a revival in the last years [18–21]: the microbial conversion of liquid C_1_ substrates, such as formic acid and methanol, referred to as C_1_ fermentation, which was already investigated in the 1970s [4, 5, 22]. Moreover, application of liquid substrates avoids also gas–liquid mass transfer issues as occurring in gas fermentation. The production of methanol or formic acid via thermo- or electro-catalysis requires only CO_2_, H_2_ (e.g. from water) and renewable energy [23]. Consequently, fermentation of such substrates enables the indirect utilization of CO_2_ as carbon and energy source in bioprocessing, which is a high-level goal for sustainable (and eventually carbon–neutral or even carbon-negative) production of chemicals and commodities [23, 24]. Hence, the application of native methylotrophs, like the gram-negative α-proteobacterium *Methylorubrum extorquens*, is a promising approach to utilize methanol for CO_2_-based production of industrially relevant chemicals and fuels [25].

*M. extorquens* is a model organism for methylotrophy and its metabolism is well understood. A genome-scale metabolic model exists to explore the capabilities of methylotrophic metabolism and to derive strain designs for metabolic engineering [26, 27]. Moreover, recent improvements of genetic tools led to a better accessibility of this microbe to alter its metabolism [28–30]. Various products were derived using this strain, e.g. l-lysine [31], α-humulene [32], 2-hydroxyisobutyric acid [33], 1-butanol [34, 35], isobutanol [36], itaconic acid [37], mesaconic acid and methylsuccinic acid [38–40], 3-hydroxypropionic acid [41] and PHA/PHB [42, 43]. However, yields and titers are still below industrial relevance and further investigations and engineering are needed to fulfill performance requirements.

Glycolic acid (GA) is a two-carbon α-hydroxy acid that is used in dyeing and tanning industry, food industry, cosmetic industry, and as a monomer or co-monomer for production of biodegradable polymers, such as polyglycolide (PGA) or poly(lactide-co-glycolide) (PLGA). Consequently, GA supports a large market that is expected to reach about 415 million US$ globally by 2024 [44]. Conventionally, GA is mainly produced from petrochemically derived formaldehyde, which is catalytically carbonylated with carbon monoxide. However, biotechnological production of GA was successfully evaluated in many studies harnessing enzymatic or fermentative conversion of various feedstock like sugars, glycerol, or ethylene glycol. Attractive process examples were published delivering yields and product titers close to industrial relevance [44]. These conversion processes derive GA by reduction of glyoxylate using glyoxylate reductases. This strategy was demonstrated by recruiting the glyoxylate shunt pathway in combination with TCA cycle balancing in *E. coli* reaching more than 65 g_GA_ L^−1^ from glucose in fed-batch fermentation with 90% of the theoretical yield (2 mol_GA_ mol_glucose_^−1^) [45]. Additionally, oxidation pathways of glycolaldehyde were investigated but showed inferior yields in comparison to the glyoxylate shunt pathway [44]. In 2021, the company Metabolic Explorer S.A. announced the first industrial fermentation process implementation delivering bio-based GA from renewable feedstock relying on engineered *E. coli* [46, 47]. This achievement shows the general viability of biotechnological production of GA, however limited to date to sugars as substrate. Hence, more sustainable and scalable approaches for the fermentative production of GA are of high interest where substrates like methanol are used.

In this study, we applied systems metabolic engineering of *Methylorubrum extorquens* TK 0001 for enhanced GA production from methanol as sole carbon and energy source. To the best of our knowledge, there are no publications reporting the test of GA production by overproducing a glyoxylate reductase in *M. extorquens* or showing native GA production by *M. extorquens* although a NADH-dependent glyoxylate reductase is annotated [26]. *M. extorquens* TK 0001 was chosen in our approach as a straight-forward host for GA production due to the occurrence of glyoxylate as an intermediate of the serine cycle that is part of the organism’s native central carbon metabolism. In addition, no plasmids are present in *M. extorquens* TK 0001 enabling a more streamlined strain engineering of this organism in comparison to the model organism *M. extorquens* AM1 [48–50].

In a first step, the potential of GA production by *M. extorquens* was evaluated in silico by establishing and analyzing a metabolic core model based on the recently published genome-scale model *i*RP911 representing *M. extorquens* AM1 [26]. In particular, elementary flux mode analysis (EFMA) was applied to gain insight into pathways that lead to maximal GA yield on methanol. An initial GA producer strain was then obtained by episomal overexpression of various glyoxylate reductase genes. Subsequently, the relevance of increasing the glyoxylate precursor pool for improving production was evaluated. This was done by glyoxylate feeding experiments and, in a second approach, by overexpression of the ethylmalonyl-CoA mutase gene, encoding a key enzyme within the ethylmalonyl-CoA pathway (EMCP), to support glyoxylate regeneration [51, 52]. Surprisingly, it was found that lactic acid (LA) is a not foreseen by-product of GA production in *M. extorquens*. The best performing producer strain was tested in fed-batch fermentation to evaluate the production performance under relevant process conditions. Finally, as an outlook, we present computed metabolic intervention strategies that would couple growth with obligate production of GA.

## Materials and methods

### Metabolic modeling

For the in silico evaluation of the potential of *M. extorquens* for GA production from methanol in silico, the genome-scale model *i*RP911 [26] was used to derive a core model of the central metabolism investigated in this study. The model comprises 144 reactions and 131 metabolites and promotes formation of biomass, carbon source (methanol and formate) uptake, exchange of O_2_, CO_2_, SO_4_^2−^, NH_4_^+^, H_2_O, and P_i_ as well as excretion of the products GA and lactic acid (LA). Reaction stoichiometries were partially adapted to obtain consistent mass and charge balances and the biomass formation reaction was corrected as described in the supplemental file S01. A heterologous NADPH-dependent glyoxylate reductase was modeled by adding the corresponding reaction R090a (in addition to the native NADH-variant R090b, [49]) to the core model.

**R090a:** 1 H^+^  + 1 glyoxylate + 1 NADPH ↔ 1 glycolate + 1 NADP^+^

Since LA formation was observed in the experiments, it was assumed to occur by the native NADH-dependent d-lactate dehydrogenase (R089, LDH_D) present in the original model *i*RP911. Another pathway potentially forming LA from methylglyoxal as precursor was modeled in silico by adding the (condensed) reaction to the core model:

**R091:** 2 H_2_O + 1 glycine + 1 acetyl-CoA + 1 O_2_ = 1 CO_2_ + 1 NH_4_^+^  + 1 CoA + 1 d-lactate + 1 H_2_O_2_ (non-reversible).

The latter reaction is based on the following reactions assumed to be present due to occurrence of annotated genes in *M. extorquens* TK 0001 and AM1 [26, 49]:

**TK0001_5800:** 1 glycine + 1 acetyl-CoA = 1 l-2-aminoacetoacetate + 1 CoA.

**Spontaneous (**[53]**):** 1 l-2-aminoacetoacetate = 1 aminoacetone + 1 CO_2_.

**TK0001_0958:** 1 aminoacetone + 1 H_2_O + 1 O_2_ = 1 methylglyoxal + 1 H_2_O_2_ + 1 NH_4_^+^

**R_0111 (*i*RP911):** 1 methylglyoxal + 1 glutathione = 1 s-lactoylglutathion.

**R_0102 (*i*RP911):** 1 s-lactoylglutathion + 1 H_2_O = 1 d-lactate + 1 glutathione.

Following Peyraud et al. [26], we assumed a non-growth associated ATP demand of 9.5 mmol_ATP_ g_CDW_^−1^ h^−1^; this value was used as lower bound for the rate of the associated ATPM_NGAM pseudo reaction. For growth rate calculations we set an upper bound for the methanol uptake rate of 15 mmol g_CDW_^−1^ h^−1^, which is close to uptake rates measured for the wild-type strain in our lab (see also below). Detailed information on the model is given in the supplemental files S01 and the model is provided in CellNetAnalyzer and SBML format on the associated GitHub repository accessible under https://github.com/JoFa-IGB/MextorquensCoreModel.git. The model was implemented and analyzed with constraint-based methods, including parsimonious FBA, flux variability analysis (FVA), elementary flux modes analysis (EFMA), and minimal cut set (MCS) analysis using the MATLAB (R2019a) software package CellNetAnalyzer (CNA) (V2023.1) [26, 54, 55]. Elementary flux modes (EFMs) were calculated by the integrated efmtool of CNA [56].

### Microorganisms and plasmids

*M. extorquens* TK 0001 DSM 1337 was used for strain engineering (German Collection of Microorganisms and Cell Cultures GmbH DSMZ, www.dsmz.de). For cloning purposes and plasmid amplification, the strain *E. coli* DH10B was obtained from Invitrogen (Darmstadt, Germany). Gene expression in *M. extorquens*, was conducted with the episomal vector pTE1887 [29, 57]. All strains and plasmids used in this study are displayed in Table 1.

**Table 1:** Summary of used strains and plasmids

Strain/plasmid	Description	References
Strain		
Mea	*M. extorquens* TK 0001 DSM 1337	www.dsmz.de, [49]
Mea-C	Mea + pTE1887 empty vector	This study
Mea-GA1	Mea + pTE1887_*ghrA*_*eco*_	This study
Mea-GA2	Mea + pTE1887_*ghrA*_*eco*_-*ecm*_*mea*_	This study
Mea-GA3	Mea + pTE1887_*ghrA*_*eco*_-*ecm*_*rsh*_	This study
Plasmid		
pTE1887	Episomal vector for gene expression in *M. extorquens* containing the ColE1 origin of replication for *E. coli*, the pMG160 sequence for replication in *M. extorquens*, empty multiple cloning site with Strep-II tag, kanamycin resistance (*Km*^*R*^), synthetic promoter P_*L/O4/A1*_ induced with IPTG	[29]
pTE1887-*ghrA*_*eco*_	pTE1887 derivative, codon-optimized *ghrA*_*eco*_, cloned with NcoI and Gibson assembly, *Km*^R^	This study
pTE1887-*pfGoxRed_1*	pTE1887 derivative, codon-optimized *pfGoxRed_1*, cloned with NcoI and Gibson assembly, *Km*^R^	This study
pTE1887-*pfGoxRed_2*	pTE1887 derivative, codon-optimized *pfGoxRed_2*, cloned with NcoI and Gibson assembly, *Km*^R^	This study
pTE1887-*pfGoxRed_3*	pTE1887 derivative, codon-optimized *pfGoxRed_3*, cloned with NcoI and Gibson assembly, *Km*^R^	This study
pTE1887-*tlitGoxRed_1*	pTE1887 derivative, codon-optimized *tlitGoxRed_1*, cloned with NcoI and Gibson assembly, *Km*^R^	This study
pTE1887-*tlitGoxRed_2*	pTE1887 derivative, codon-optimized *tlitGoxRed_2*, cloned with NcoI and Gibson assembly, *Km*^R^	This study
pTE1887-*pfuGoxRed*	pTE1887 derivative, codon-optimized *pfuGoxRed*, cloned with NcoI and Gibson assembly, *Km*^R^	This study
pTE1887-*sceGoxRed*	pTE1887 derivative, codon-optimized *sceGoxRed*, cloned with NcoI and Gibson assembly, *Km*^R^	This study
pTE1887-*tthGoxRed*	pTE1887 derivative, codon-optimized *tthGoxRed*, cloned with NcoI and Gibson assembly, *Km*^R^	This study
pTE1887-*ghrB*_*eco*_	pTE1887 derivative, codon-optimized *ghrB*_*eco*_, cloned with NcoI and Gibson assembly, *Km*^R^	This study
pTE1887-*meaGoxRed*	pTE1887 derivative, native *meaGoxRed* from *M. extorquens* TK 0001, cloned with NcoI and Gibson assembly, *Km*^R^	This study
pTE1887-*meaGoxRed_ATG*	pTE1887 derivative, *meaGoxRed* from *M. extorquens* TK 0001 with exchange of start codon to ATG, cloned with NcoI and Gibson assembly, *Km*^R^	This study
pTE1887-*aaceGoxRed_1*	pTE1887 derivative, codon-optimized *aaceGoxRed_1*, cloned with NcoI and Gibson assembly, *Km*^R^	This study
pTE1887-*aaceGoxRed_2*	pTE1887 derivative, codon-optimized *aaceGoxRed_2*, cloned with NcoI and Gibson assembly, *Km*^R^	This study
pTE1887-*aaceGoxRed_3*	pTE1887 derivative, codon-optimized *aaceGoxRed_3*, cloned with NcoI and Gibson assembly, *Km*^R^	This study
pTE1887-*ghrA*_*eco*_-*ecm*_*mea*_	pTE1887-*ghrA*_*eco*_ derivative, native *ecm*_*mea*_, cloned with BamHI and Gibson assembly, *Km*^R^	This study
pTE1887-*ghrA*_*eco*_-*ecm*_*rsh*_	pTE1887-*ghrA*_*eco*_ derivative, *ecm*_*rsh*_, cloned with BamHI and Gibson assembly, *Km*^R^	This study

The genes of various glyoxylate reductases and *ecm*_*rsh*_ (ethylmalonyl-CoA mutase from *Rhodobacter sphaeroides* ATCC 17029) were ordered as *M. extorquens* codon-optimized genes from BioCat GmbH (Heidelberg, Germany)

### DNA manipulation and strain construction

Standard molecular techniques were applied for amplification, purification, and transformation of DNA [58]. Cloning of DNA into the pTE1887 vector was conducted using Gibson assembly subsequent to plasmid linearization with the indicated restriction enzymes [59]. In addition, *ecm*_*mea*_ (ethylmalonyl-CoA mutase from *M. extorquens* TK 0001 DSM 1337) was isolated with PCR from the genome using the primer pair (fw_*ecmmea*_pTE1887/rv_*ecmmea*_pTE1887). The final vectors were transformed into *M. extorquens* by electroporation [60]. Routinely, strain construction was verified by colony PCR and plasmid sequencing (Eurofins Genomics Germany GmbH, Ebersberg, Germany) using the primer pair 2698 and 2430, respectively. The sequences of used oligonucleotides and codon-optimized genes can be found in the supplementary file S02 in Table S1 and Table S2.

### Strain maintenance

Routinely, liquid cultivation of recombinant *E. coli* strains for cloning and plasmid amplification was conducted using Lysogeny broth medium containing 10 g L^−1^ tryptone, 5 g L^−1^ yeast extract, 10 g L^−1^ NaCl, and 30 µg mL^−1^ kanamycin for plasmid selection. To obtain agar plates, 15 g L^−1^ Agar Kobe I (Carl Roth, Karlsruhe, Germany) was added.

Minimal medium (MO) for aerobic cultivation of *M. extorquens* strains was always prepared freshly from sterile stock solutions and sterile demineralized water as described previously [61]. Methanol was used as sole carbon source in indicated concentrations. For strain propagation and clone selection, the medium was mixed with 18 g L^−1^ Agar Kobe I, 1% methanol (v/v) and 30 µg mL^−1^ kanamycin. Inoculated agar plates were incubated at 30 °C until clearly visible single colonies could be obtained. Exponentially growing cells were harvested and frozen at -80 °C as cryo stocks with a final concentration of 30% (v/v) glycerol for strain maintenance.

### Batch cultivation in shake flasks

Aerobic cultivation studies in MO were conducted in 250 mL baffled shake flasks (DURAN^®^, DWK Life Sciences, Wertheim, Germany) at 150 rpm and 30 °C in a humidified atmosphere (Innova 44, diameter 2.5 cm, Eppendorf AG, Hamburg, Germany). In order to screen functional glyoxylate reductases and obtain biomass for crude cell extracts to conduct enzyme assays, the following seed train was applied. To obtain a viable preculture, 50 mL medium with 1% methanol (v/v) and 30 µg mL^−1^ kanamycin, was inoculated from a cryo stock and incubated for 68 h. The preculture was used to inoculate 50 mL medium with 1% methanol (v/v) and 30 µg mL^−1^ kanamycin to an initial OD_600_ of 0.1 and incubated for 30 h. Finally, main cultures of 50 mL MO containing 1% methanol (v/v) and 30 µg mL^−1^ kanamycin were inoculated to an initial OD_600_ of 0.05. Expression of glyoxylate reductases was induced by addition of 1 mM IPTG at OD_600_ of 1.0. Samples for HPLC analysis and biomass for enzyme assays were harvested 40 h after inoculation.

To analyze microbial growth, substrate consumption, and product formation in detail, the following procedure was routinely used. To obtain a viable preculture, 25 mL MO containing 1% methanol (v/v) and 30 µg mL^−1^ kanamycin were inoculated from cryo stock and incubated for 30 h to an OD_600_ of 4–6. Subsequently, main cultures of 50 mL MO containing 1% methanol (v/v) and the respective antibiotic were inoculated to an initial OD_600_ of 0.08. Cells were cultivated overnight before gene expression was induced at OD_600_ of 1.0 by adding a final concentration of 1 mM IPTG. Additional cultivations were performed with supplementation of a final concentration of 1 g L^−1^ glyoxylate (pH neutral) which was added to the main cultures 1 h after induction. All main cultures were conducted in 250 mL baffled screw cap flasks and adequate biological replicates as indicated.

To monitor microbial growth, regular samples of 1–2 mL were withdrawn from cultures. OD_600_ and pH were measured directly. Cells were removed by centrifugation and the supernatant was used for substrate and product quantification by HPLC analysis.

### Batch cultivation in parallelized microbioreactors

Native product tolerance of *M. extorquens* TK 0001 was investigated by cultivating the strain TK 0001 + pTE1887 with gradually increasing GA concentrations in a high-throughput experiment using a parallelized and miniaturized microbioreactor system (1–2 mL scale) (BioLector, m2p-labs GmbH, Baesweiler, Germany).

The preculture of TK 0001 + pTE1887 was inoculated in 25 mL MO containing 1% (v/v) methanol and 30 μg mL^−1^ kanamycin using several colonies from a MO agar plate. The main cultures were inoculated in 1 mL MO to an initial OD_600_ of about 0.1 using exponentially growing cells. The medium contained 1% (v/v) methanol, 30 μg mL^−1^ kanamycin, 1 mM IPTG and the indicated final GA concentrations to determine the tolerance limit. GA was added from pH neutral stock solution of 120 g L^−1^. Standard conditions without GA were applied as positive control.

Cultivation was conducted at 1000 rpm and 30 °C under atmospheric air composition (20.95% O_2_) and with humidity set to 85%. Cell density was recorded by acquisition of the backscatter signal at 620 nm. All main cultivations were performed in biological triplicates.

### Fed-batch cultivation in bioreactors

Fed-batch cultivation was operated in 2 L lab-scale bioreactors (BIOSTAT® B Plus, B. Braun Biotech, Berlin, Germany) initially filled with 700 mL MO supplemented with 1% methanol (v/v). The pH of the medium was monitored using a pH electrode (EasyFerm Bio HB K8 120, Hamilton, Höchst, Germany) and maintained at 6.8 ± 0.1 using 2 M sulfuric acid. The dissolved oxygen level (pO_2_) was measured using a pO_2_ electrode (OxyFerm FDA 120, Hamilton, Höchst, Germany). The pO_2_ was kept constant at 20% by applying a cascade control adapting first the agitation speed and secondly the aeration rate. Initially, agitation was set to 250 rpm (max. 1000 rpm) with an aeration rate of 0.06 sL h^−1^ (max. 3 sL h^−1^). The temperature was kept at 30 °C ± 0.2 °C. Data acquisition and process control were maintained by BIOSTAT software.

The bioreactor was inoculated to an initial OD_600_ of 0.1 using exponentially growing cells of an overnight preculture. The preculture was grown in 50 mL MO supplemented with 1% methanol (v/v) using a single colony from MO agar plate. Microbial growth, substrate consumption and product formation were monitored as described for shake flask experiments. To avoid carbon depletion, the methanol concentration was measured at-line and 5 g_MeOH_ L^−1^ were added repeatedly during the feeding phase when methanol concentration has fallen below 1 g L^−1^.

### Analytical methods

#### Determination of cell and biomass concentration

The optical density (OD_600_) at 600 nm (Ultrospec^®^ 10 (RS232), Biochrom Ltd., Cambridge, United Kingdom) was used as a measure for cell concentration. The cell dry weight (CDW) was inferred from the optical density using the correlation factor of 0.305 g_CDW_ L^−1^ OD_600_^−1^ that was determined by weighing dried biomass samples obtained from exponentially growing cells.

#### Analysis of in vivo enzyme activities

Crude cell extracts for the glyoxylate reductase enzyme assay were obtained from exponentially growing cells (OD_600_ = 6–9). Biomass was harvested by centrifugation (4,122x *g*, 15 min at 4 °C) and washed using 20 mL 50 mM Tris–HCl (pH 7.5). The cell pellet was than resuspended in 50 mM MOPS buffer (pH 6.6, 7 mL g_Wet Cells_^−1^). Subsequently, cell disruption was conducted using French Press at 1,400 bar with 2 mL samples. Finally, the extract was cleared by centrifugation (21,500x *g*, 15 min at 4 °C) followed by Nanodrop (Thermo Scientific^TM^ NanoDrop^TM^ Lite Spectrophotometer, Waltham, MA, USA) protein content determination. The enzyme assay was carried out in a plate reader at 37 °C in 200 µL total volume with 160 µL appropriately diluted cell lysate, 20 µL 50 mM glyoxylate and 20 µL 2 mM NAD(P)H. Enzyme activity was determined photometrically through measurement of change in absorption at 340 nm, caused by NAD(P)H consumption. One unit was defined as the amount of enzyme which converts 1 µmol substrate per minute.

#### Metabolite qualification and quantification

HPLC analysis for methanol, GA, and LA quantification was performed with a Prominence UFLC System from Shimadzu Corporation (Kyoto, Japan) consisting of the system controller unit CBM-20A, the solvent delivery unit LC-20AD, the degassing unit DGU-20A3, the auto sampler unit SIL-20ACHT, the column oven unit CTO-20AC and the refractive index detector unit RID-10A. System control and data evaluation was performed using the associated software LabSolutions V5.92.

Sample matrix separation was conducted with an isocratic flow rate of 0.5 mL min^−1^ for 25 min on a Synergi™ 4 µm Hydro-RP 80 Å, 250 × 4.5 mm LC column (Phenomenex Ltd. Deutschland (Aschaffenburg, Deutschland)) at 30 °C with 20 mM potassium phosphate (titrated to pH 1.5 with H_3_PO_4_, HPLC Grade) as mobile phase. Data acquisition was done using refractive index detection (RID) which was operated in analytical mode at 2 Hz for 500 ms with positive polarity at a cell temperature of 40 °C. External standard mixtures of methanol, GA, and LA were used for calibration.

Analysis of secreted products within the aqueous cultivation samples was conducted by GC/MS using a GCMS-QP2010 SE, GC-2010 Plus gas chromatograph with SGE BPX5-column (0.25 mm × 30 m), auto injector AOC-20i and auto sampler AOC-20 s from Shimadzu Corporation (Kyoto, Japan). Products were identified by comparing processed samples with external standards of the respective compound.

In short, 70 µL of a culture supernatant or an aqueous standard stock solution (100 mg L^−1^) was freeze-dried. The obtained residue was re-dissolved in 70 µL *N*,*N*-dimethyl formamide (0.1% pyridine), mixed with 70 µL *N*-methyl-*N*-t-butyl-dimethylsilyl-trifluoroacetamide (MBDSTFA) (Macherey–Nagel, Düren, Germany) and incubated for 30 min at 80 °C for complete conversion into the corresponding t-butyl-dimethylsilyl (TBMDS) derivative [62]. A subsequent centrifugation at 9.600x *g* was conducted for 3 min at 25 °C to avoid insoluble contaminants prior to measurement.

The instrument was operated at a carrier gas flow of 1.7 mL min^−1^ and temperatures of 250 °C (inlet), 230 °C (interface), and 150 °C (quadrupole). The following temperature profile was applied for separation: 120 °C for 2 min, a ramp of 8 °C min^−1^ up to 200 °C, and then 10 °C min^−1^ up to 320 °C. The samples were injected in split mode with a split ratio of 10.0. The data sets were acquired within the *m/z* range 50 – 750, in scan mode with 0.3 s scan time.

#### Determination of KPIs and LD_50_ for strain characterization

The KPIs titer (g_Compound_ L^−1^), yield Y (g_Compound_ g_Compound_^−1^), specific rate q (g_Compound_ g_CDW_^−1^ h^−1^), and volumetric rate (g_Compound_ L^−1^ h^−1^) were determined for the observed growth phases as mean of biological replicates using standard methods [63]. For cultivations supplemented with glyoxylate, the calculation of a specific carbon uptake rate q_C_ was necessary. Briefly, q_C_ was determined using Eq. 1 with *n*_MeOH_ and *n*_Gox_ being the number of carbon atoms present per molecule of methanol and glyoxylate, respectively.

1 $${\text{q}}_{{\text{C}}} = {\text{ q}}_{{{\text{S}},{\text{ MeOH}}}} \, \cdot \,n_{{{\text{MeOH}}}} + {\text{ q}}_{{{\text{S}},{\text{Gox}}}} \, \cdot \,n_{Gox}$$

In case of cultivations supplemented with glyoxylate, Y_X/S_ and Y_P/S_ were calculated based on ${\text {q}}_{\text{C}}$ instead of ${\text {q}}_{\text{S}}$.

The LD_50_ (Y(X)), corresponding to the GA concentration leading to a half-maximal growth rate µ_max_ during the tolerance tests, was determined by plotting the calculated µ_max_ against the GA concentration and fitting the data points using a Hill-like function as shown in Eq. 2.

2 $${\text{Y}}\left( {\text{X}} \right) = \,\left( {{\text{A}}^{{\text{C}}} \, \cdot \,\left( {{\text{B}}^{{\text{C}}} + {\text{X}}^{{\text{C}}} } \right)^{ - 1} } \right)\, + \,{\text{D}}$$

## Results and discussion

### Modeling of methanol-based GA production in *M. extorquens*

In order to evaluate the potential of *M. extorquens* to produce GA from methanol, a core model of the central metabolism of *M. extorquens* was constructed (see Methods) and analyzed with techniques of constraint-based modeling. A map of the model is shown in Fig. 2.

#### Prediction of maximal growth rate of wild-type strain

As a first step, we used parsimonious FBA to compute the maximal growth rate on methanol yielding 0.23 h^−1^. The resulting flux distribution supporting maximal growth rate is shown in Figure S1 in Supplemental File S02. The maximal growth rate is reached at the assumed upper boundary of the methanol uptake rate (15.0 mmol_MeOH_ g_CDW_^−1^ h^−1^) and corresponds to a biomass yield of 0.479 g_CDW_ g_MeOH_^−1^. The model-predicted growth rate is slightly higher than wild-type growth rates (0.18–0.2 h^−1^) of *M. extorquens* TK 0001 measured in our lab, which were associated with methanol uptake rates of 15.0–15.7 mmol_MeOH_ g_CDW_^−1^ h^−1^. With this, also the predicted maximal biomass yield Y_X/MeOH_ is moderately higher (ca. 20%) than our (and previously reported [26]) values. One possible reason for the overestimated growth yield could be an underestimated energy demand, either in the assumed non-growth associated ATP demand (ATPM_NGAM; 9.5 mmol_ATP_ g_CDW_^−1^ h^−1^) or in the growth-associated ATP demand of 59.81 mmol g_CDW_^−1^, which was originally taken from *E. coli* [26]. Additionally, as will be further discussed below, there are two main methanol oxidation routes (periplasmic and cytoplasmic, [64]), which deliver both formate as the entry metabolite for carbon assimilation but result in different ATP yields and thus different biomass yields. In both cases, the generated formate is condensed with ATP and tetrahydrofolate (THF) to 10-formyltetrahydrofolate that is in turn converted to methylenetetrahydrofolate (MLTHF) by Formate-tetrahydrofolate ligase (R007) [65]. MLTHF is then reduced using NADPH to methenyltetrahydrofolate (METHF) by MLTHF dehydrogenase (R015), which is next condensed with glycine to l-serine by Glycine hydroxymethyltransferase (R016) entering serine cycle (Figure S1 in Supplemental File S02). The described interplay of THMPT (cytoplasmic conversion of formaldehyde to formate) and THF-dependent reactions is from now on referred to as the THMPT/THF-node.

Despite the moderate overestimation of biomass yield, we consider the model sufficient for our purposes mainly focusing on product synthesis (see also below). The model also clearly predicts an essential requirement of oxygen for growth (Figure S3 in Supplemental File S02), which is in line with the fact that *M. extorquens* is a strictly aerobic bacterium [26, 66, 67].

#### Elementary flux mode analysis: synthesis of ATP

Next, we calculated and analyzed the elementary flux modes (EFMs) of the model with respect to three objectives: (i) net formation of ATP, (ii) GA synthesis (without growth) and (iii) growth-coupled GA formation. For the latter two objectives the use of either NADPH- or NADH-dependent glyoxylate reductase was considered. For each objective, the number of EFMs and the maximum achievable theoretical yields were derived (Table 2).

**Table 2:** EFMs of the constructed core model of *M. extorquens*

Objective	#EFMs	Maximal yield (#EFMs with maximal yield)
**ATP** (w/o GA production)	90,915	${Y}_{ATP/MeOH}^{max}$= 5 mol_ATP_ mol_MeOH_^−1^ (6 EFMs)
**GA production** thereof: **NADPH-dependent GR** **NADH-dependent GR**	267,347 129,753 137,594	${Y}_{GA/MeOH}^{max}$= 0.500 mol_GA_ mol_MeOH_^−1^ (320 EFMs) ${Y}_{GA/MeOH}^{max}$ = 0.500 mol_GA_ mol_MeOH_^−1^ (108 EFMs) ${Y}_{GA/MeOH}^{max}$ = 0.500 mol_GA_ mol_MeOH_^−1^ (212 EFMs)
**GA with growth** thereof: **NADPH-dependent GR** **NADH-dependent GR**	226,240 111,278 114,962	${Y}_{GA/MeOH}^{max}$= 0.470 mol_GA_ mol_MeOH_^−1^ (4 EFMs) **→** Max. biomass yield at max. GA yield: ${Y}_{X/MeOH}$ = 0.042 g_CDW_ g_MeOH_^−1^ ${Y}_{GA/MeOH}^{max}$ = 0.487 mol_GA_ mol_MeOH_^−1^ (4 EFMs) **→** Max. biomass yield at max. GA yield: ${Y}_{X/MeOH}$ = 0.018 g_CDW_ g_MeOH_^−1^

The EFMs were grouped and maximum yields (Y^max^) calculated for the three objectives: net ATP formation, GA production without growth, and growth-coupled GA production. EFMs involving GA synthesis considered either the NADPH- or the NADH-dependent glyoxylate reductase (GR)

Regarding ATP synthesis, we found 90,915 modes that supported net ATP production from methanol with 6 modes exhibiting the maximal ATP yield of 5 mol_ATP_ mol_MeOH_^−1^, matching results of the original genome-scale model [26]. In all of these modes methanol is exclusively oxidized to CO_2_ via cytoplasmic FDH. An example of such a flux distribution is given in Figure S2 in Supplemental File S02. The cytoplasmic condensation of formaldehyde with tetrahydromethanopterin (THMPT) to methylenetetrahydromethanopterin (MLTHMPT) prior to oxidation to formate enables flexible NAD(P)H formation. The latter is fulfilled by the NADPH/NADH-dependent MLTHMPT dehydrogenase (R009 and R010) releasing methenyltetrahydromethanopterin (METHMPT) and a first NAD(P)H. The cytosolic end product formate is then oxidized to CO_2_ by cytoplasmic FDH releasing a second NADH. No NADPH-dependent FDH is contained in our (or the previous genome-scale) model. Consequently, under maximal production of 5 mol ATP per mol methanol, either 2 mol NADH and 0 mol NADPH or 1 mol NADH and 1 mol NADPH are produced (see Figure S2 in Supplemental File S02). However, the formation of 2 mol NADPH is only possible with an NADPH-dependent FDH whose existence in the organism remains speculative [68].

In contrast to the EFMs with cytoplasmic FDH, there is a single EFM using the route of periplasmatic methanol oxidation, which delivers only 3 instead of 5 mol_ATP_ mol_MeOH_^−1^. The reason for the reduced ATP yield in periplasmic oxidation is that no net NAD(P)H is generated due to the loss of electrons via reduced cytochrome-c from which the electrons are then transferred to oxygen as final acceptor [26]. To the best of our knowledge, it is so far unknown in literature whether the periplasmic route is active in vivo or if a distinct flux split between both routes exists for fine tuning energy and redox requirements of methylotrophically grown cells [26, 64, 69, 70]. Importantly, a contribution of periplasmic oxidation with lower ATP yield could be the reason for the observed lower growth rates and biomass yields compared to the model prediction. Indeed, if exclusively periplasmic oxidation route is allowed in the model, a maximal growth rate of 0.176 h^−1^ (Y_X/MeOH_ of 0.366 g_CDW_ g_MeOH_^−1^) is predicted, which strikingly matches our or previously measured in vivo data. In conclusion, we hypothesize that the periplasmic route may significantly contribute to C_1_ assimilation.

#### Elementary flux mode analysis: synthesis of GA

**Fig. 1:**
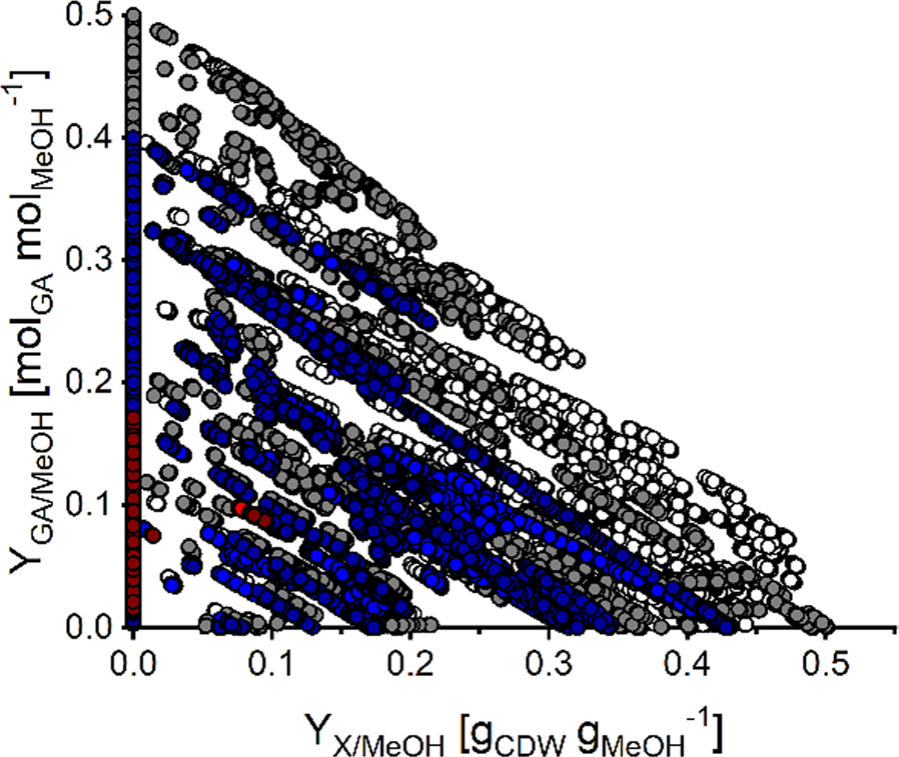
Yield-space analysis based on EFMs with GA production. EFMs with GA production of the core network with NADPH- or NADH-dependent glyoxylate reductase (white and grey circles, respectively) were plotted with respect to their biomass and GA yields (Y_X/MeOH_ and Y_GA/MeOH_). Effects of genetic perturbations on yield space calculations were simulated by in silico deletion of formate dehydrogenases (FDHs; non-affected EFMs shown by blue circles) and phosphoenolpyruvate carboxylase (PPC; non-affected EFMs shown by red circles) reactions. To simulate the reaction deletion, only EFMs were selected which do not use the corresponding reaction (zero flux)

Next, we analyzed the EFMs that lead to production of GA. In total, 312,373 EFMs with GA formation were calculated. To prevent analysis of EFMs with unrealistic futile cycling between glyoxylate and glycolate, all EFMs containing parallel activity of NADPH- and NADH-dependent glyoxylate reductase were excluded (45,026 EFMs). Consequently, a set of 267,347 EFMs with GA formation was analyzed (Fig. 1).

**Fig. 2:**
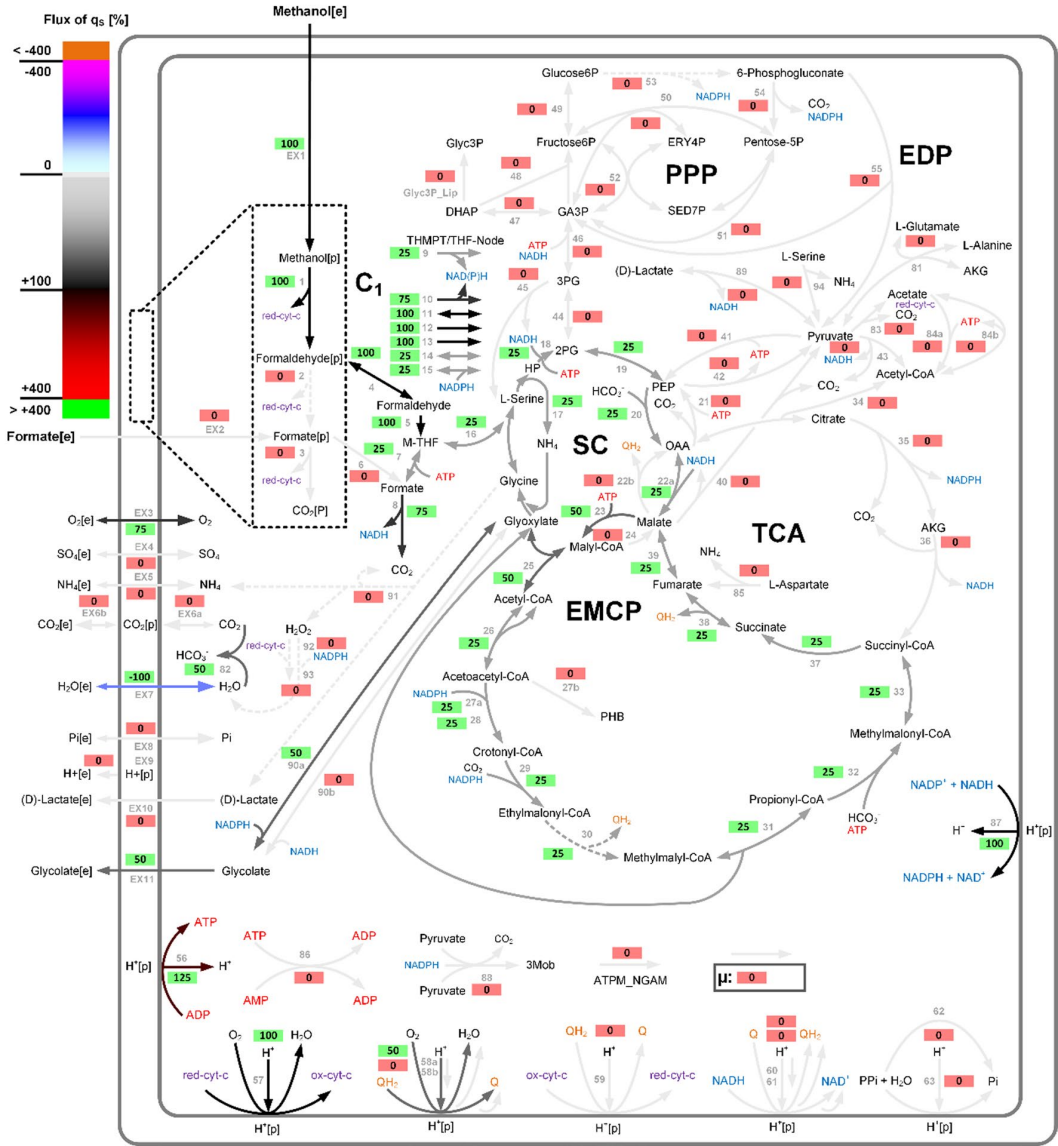
Map of the central carbon metabolism of the *Mextorquens* CoreModel displaying an EFM with maximal GA yield of 0.5 mol_GA_ mol_MeOH_^−1^. Shown are exchange of metabolites, methanol and formate uptake and oxidation, C_1_-interconversions (THMPT/THF-node), Serine Cycle (SC), Citric Acid Cycle (TCA), Ethylmalonyl-CoA Pathway (EMCP), Pentose Phosphate Pathway (PPP), Entner-Doudoroff Pathway (EDP), respiratory processes, transhydrogenase, ATP generation, growth rate µ, non-growth-associated ATP demand (ATPM_NGAM), and a compressed methylglyoxal pathway for LA formation. The gradual arrow color changes indicate the fluxes in % relative to methanol uptake (see Figure Legend). Green boxes indicate active reactions (and their flux value) and red boxes inactive reactions. A summary of abbreviations of metabolites is given in supplement file S01

The maximal theoretical GA yield is Y_GA/MeOH_ = 0.5 mol_GA_ mol_MeOH_^−1^ (or 1.19 g_GA_ g_MeOH_^−1^) corresponding to a 100% carbon yield (1.0 C-mol_GA_ C-mol_MeOH_^−1^). This maximal GA yield is exhibited by 320 EFMs, some of which use the NADPH- and some the NADH-dependent glyoxylate reductase. Figure 2 displays an exemplary EFM with the maximal GA yield of 0.5 mol_GA_ mol_MeOH_^−1^. The latter distribution involves methanol uptake and subsequent oxidation, formate dissimilation to CO_2_. Formate assimilation is driven by the serine cycle via formaldehyde and the THMPT/THF-node and the central serine cycle. The EMCP is used for replenishing glyoxylate. Finally, glyoxylate is reduced by NADPH-dependent glyoxylate reductase. Remarkably, transhydrogenase is used for NADPH generation. The respiratory processes are applied for redox balancing of cytochrome-c and the ubiquinone pool. Generally, all yield-optimal EFMs have the following net stoichiometry of Eq. (3):

3 $${\text{2 CH}}_{{3}} {\text{OH}}\, + \,{1}.{\text{5 O}}_{{2}} \, \to \,{\text{C}}_{{2}} {\text{H}}_{{3}} {\text{O}}_{{3}}^{ - } \, + \,{2}\,{\text{H}}_{{2}} {\text{O}}\, + \,{1}\,{\text{H}}^{ + }$$

The carbon-based yield of 1.0 C-mol_GA_ C-mol_MeOH_^−1^ indicates that highly efficient production of GA by *M. extorquens* is possible due to a 100% carbon yield without carbon loss in the form of CO_2_ (see Fig. 2 and below). Crucial for this carbon yield is balancing carbon dissimilation (CO_2_-release via FDH) and fixation via the CO_2_- or bicarbonate-dependent reactions of phosphoenolpyruvate carboxylase (PPC) in the serine cycle as well as crotonyl-CoA carboxylase/reductase (CCR) and propionyl-CoA-Carboxylase (PCC) in the EMCP. In particular, it can be shown that individual in silico deletion of PPC or both FDHs reduces Y_GA/MeOH_ substantially to 0.4 (∆PPC) or even below 0.2 mol_GA_ mol_MeOH_^−1^ (∆FDHs), pinpoint the importance of a balanced carbon dissimilation (Fig. 1). Moreover, all GA-producing EFMs require activity of the EMCP. We also found that, with NADPH as cofactor for the glyoxylate reductase, all EFMs with optimal GA yield (108 EFMs) required activity of the transhydrogenase reaction (R087) to satisfy the needs of NADPH for glyoxylate reduction to GA. Only 8 EFMs recruited in addition the oxidative glucose 6-phosphate dehydrogenase (R053) as entry for the oxidative pentose phosphate pathway. Additional NADPH expenses are related to the EMCP enzymes acetoacetyl-CoA reductase (R027a) and crotonyl-CoA carboxylase/reductase (CCR) (R029). In contrast, transhydrogenase reaction was not necessarily required to achieve highest GA yield when applying NADH-dependent glyoxylate reductase. In this case, the NADPH and NADH demands were completely balanced by THMPT/THF-node reactions of MLTHMPT dehydrogenase (R009 and R010), and in addition NADH-dependent formate dehydrogenase (FDH). Another interesting observation was that some EFMs with the maximal GA yield of 0.5 mol_GA_ mol_MeOH_^−1^ can even co-produce ATP (up to 0.188 or 0.250 mol_ATP_ mol_MeOH_^−1^ with NADPH- or NADH-dependent glyoxylate reductase, respectively).

As indicated in Eq. 3, 1.5 mol O_2_ per mol GA are required for maximal GA yield. Due to the reactions of formaldehyde oxidation to formate (either within the THMPT reaction cascade or via the periplasmic formaldehyde oxidation to formate), the oxygen atom originates from H_2_O and not directly from molecular oxygen. As shown in Fig. 2, the oxygen taken up is primarily required for respiratory oxidation, thus for regeneration of cytochrome-c and ubiquinone (R057 and R058a and R058b), and one third of the H_2_O produced in these reactions is eventually involved in carrying over the additional oxygen atom contained in GA (three oxygen atoms in GA vs. two oxygen atoms from two molecules of methanol). Generally, we found that all GA-forming EFMs require at least 1.5 mol O_2_ per mol GA. Last but not least, the overall reaction shown in Eq. 3 has a standard free energy of ∆_r_G^’0^ = -727.6 kJ mol^−1^ (calculated with eQuilibrator [71]), indicating a high thermodynamic support of methanol-based GA production in *M. extorquens*.

It is important to note that, in contrast to the found optimal GA pathways (EFMs) in *M. extorquens* exhibiting 100% carbon efficiency, sugar-based pathways support only a carbon atom efficiency of up to 66% [44]. However, a new synthetic pathway, named glycoptimus, was presented recently for *E. coli* that is potentially capable to yield also up to 1 C-mol GA per C-mol pentose or hexose (up to 1.27 g g^−1^) via maximal carbon conservation [72]. The glycoptimus pathway recruits pentose phosphate pathway interconversions to form glycolaldehyde without loss of carbon into CO_2_. Unfortunately, the implementation of glycoptimus into *E. coli* enabled Y_GA/S_ of 0.19 mol_GA_ mol^−1^ (glucose) to 0.68 mol_GA_ mol^−1^ (arabinose), representing only 6% to 27% of the calculated theoretical maximum of the glycoptimus pathway, respectively.

Consequently, we assume that the GA production potential of *M. extorquens* is superior to conventional glycolytic producer strains and at least comparable to the glycoptimus pathway. The key for high-yield product formation is the serine cycle intermediate glyoxylate and its regeneration by the EMCP and coupled CO_2_ (or bicarbonate) fixation [26]. With this in mind, it seems also promising to apply heterologous phosphoenolpyruvate carboxykinase (PCK) to connect oxaloacetate supply with extra ATP formation in *M. extorquens* instead of dissipating the energy in form of organic phosphate generated by PEP carboxylase. A similar approach was used for high yield succinic acid production in *E. coli* [73]. Suitable microbial genes of PCK candidates are available and could be used in future studies [74, 75]. However, this approach might be limited due to thermodynamic constraints and likely only possible at low ATP concentrations.

We finally investigated EFMs where GA synthesis is coupled with growth. Those EFMs are of particular relevance as they demonstrate the feasibility of developing (knock-out) strains in which the product GA is an obligatory byproduct of growth (growth-coupled production) [76]. As shown in Table 2, there are many growth-coupled EFMs, and even at high yields, only a small fraction of carbon is diverted to biomass synthesis (18 to 42 mg_CDW_ g_MeOH_^−1^), which still allows high GA yields close to the maximum (0.487 mol_GA_ mol_MeOH_^−1^; Table 2, Fig. 1). In a later “Intervention strategies enforcing growth-coupled GA synthesis” section, we will compute intervention strategies (minimal cut sets) that enforce growth-coupled synthesis of GA.

Generally, the obtained modeling results strikingly support that *M. extorquens* provides a high potential to achieve efficient GA production. Sufficient oxygen supply and CO_2_ levels during the fermentation as well as provision of sufficient amounts of redox cofactors are predicted as key factors to support enhanced GA formation.

### *M. extorquens* tolerates increased GA amounts in batch fermentation

In advance to engineering *M. extorquens* for GA production, it was evaluated if the strain tolerates elevated product concentrations. For that purpose, a microbioreactor cultivation was conducted using *M. extorquens* TK 0001 + pTE1887 (Mea-C) in standard minimal medium supplied with 1% (v/v) methanol and increasing concentrations of GA.

**Fig. 3:**
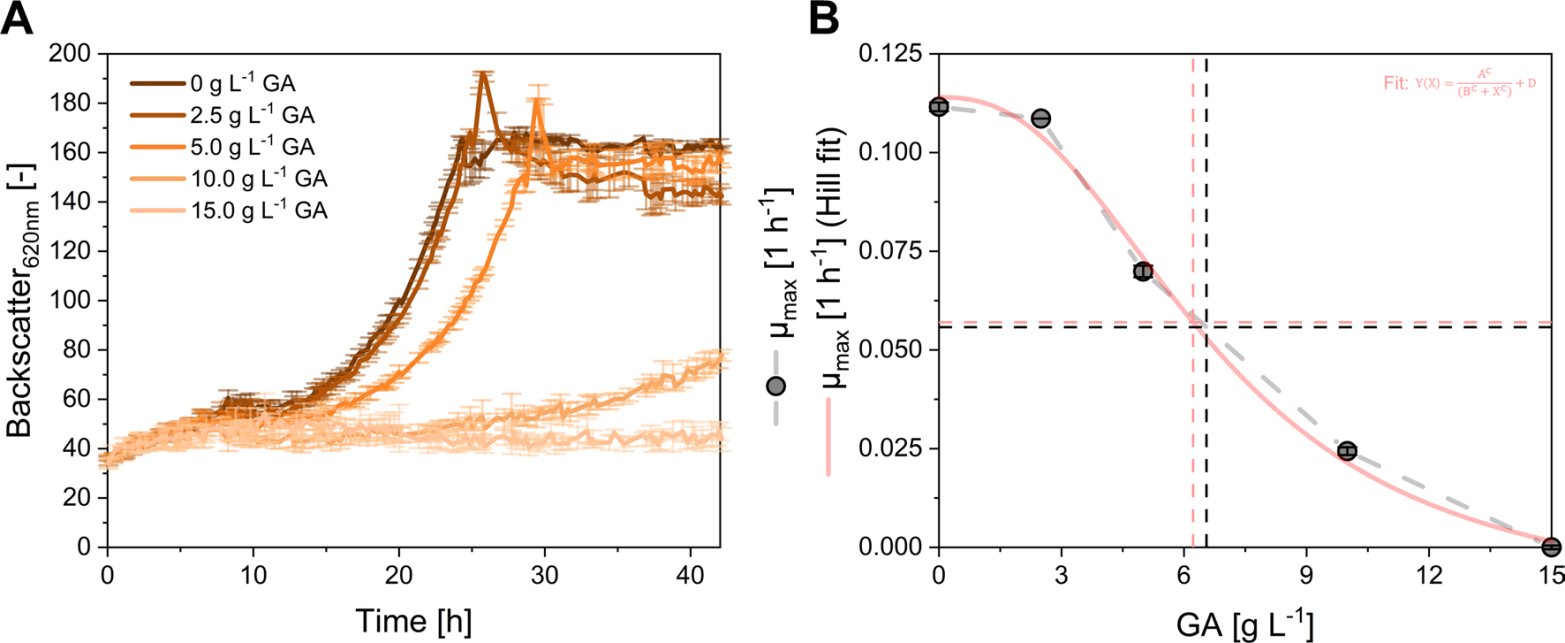
Product tolerance of *M. extorquens* TK 0001 + pTE1887 (Mea-C) in microbioreactor cultivations. **A** Growth profiles of Mea-C in the BioLector experiments applying GA concentrations in the range between 0 and 15 g L^−1^. **B** Inhibition curve of growth rate µ in relation of the applied GA concentrations. Data and standard deviation represent three independent biological replicates (n = 3)

After a prolonged lag phase of about 10 h, the cells started growing exponentially. While the *M.* *extorquens* strain grown without GA supply showed unaffected growth as expected, the growth rate decreased with addition of increasing GA concentration (Fig. 3A).

When 2.5 to 5.0 g L^−1^ GA was supplied a decrease of µ_max_ by 2.6% to 37.5% compared to reference condition without GA was observed. When 10 g L^−1^ GA were added to the medium, µ_max_ was substantially hampered by about 80% and growth ceased completely when 15 g L^−1^ GA were added. By fitting the data points to a Hill like equation, the concentration leading to a reduction of µ_max_ by 50% (LD_50_) was determined to be 6.22 g L^−1^ of GA (Fig. 3B, red line) [77]. When the data points were manually connected (Fig. 3B, grey dashed lines) the LD_50_ was with 6.55 g L^−1^ in a comparable range.

Consequently, *M. extorquens* TK 0001 tolerates up to 10 g L^−1^ GA. Microbial tolerance against substrates and products is of importance for superior cell factories to withstand gradually increasing product concentrations, or high substrate concentrations in batch phases during fermentation [78]. A comprehensive comparison of microbial robustness against important chemical products was recently conducted using *E. coli* [79]. In the latter study, it was found that e.g. acetic acid, levulinic acid, itaconic acid, and succinic acid were tolerated in the range from 4.2 g L^−1^ (acetate), to 32 g L^−1^ (succinic acid). The GA concentration of up to 10 g L^−1^ tolerated by *M.* *extorquens* TK 0001 is an acceptable starting point for an initial producer strain. However, in future strain engineering approaches the strain robustness in respect to increased GA concentrations can be targeted by adaptive laboratory evolution (ALE) or rational tolerance engineering. These strategies proved to be viable to engineer *M. extorquens* to withstand increased 1-butanol (~ 4 g L^−1^) or methanol (~ 40 g L^−1^) titers [35, 80, 81].

### Engineering of an initial *M. extorquens* GA producer strain

To unlock the capability of *M. extorquens* to produce GA from methanol, 14 glyoxylate reductase genes stemming from various host organisms were successfully expressed applying the pTE1887 vector system. The candidate genes were chosen based on BLAST analysis of the *M. extorquens* TK 0001 gene sequence TK0001_6029 against the KEGG database. The BLAST results considered several *phyla* to enhance screening success but also included previously published glyoxylate reductases [82–87]. In particular, codon-optimized genes were cloned from *Escherichia coli* K12 MG1655, *Pseudomonas fluorescens* Pf0-1, *Thermococcus litoralis*, *Pyrococcus furiosus* DSM 3638, *Saccharomyces cerevisiae*, *Thermus thermophilus* HB27, and *Acetobacter aceti*. In addition, the native gene of *Methylorubrum extorquens* TK 0001 (*meaGoxRed*) was cloned with the native start-codon TTG, or the potentially stronger start-codon ATG namely *meaGoxRed_ATG*.

Following strain construction, the screening was conducted in shake flask regimes in mineral salts medium. GA formation and glyoxylate reductase activity was quantified 19–21 h after induction using HPLC analysis and enzymatic assays, respectively. In order to evaluate cofactor dependencies, NADPH and NADH were tested individually in the assay using glyoxylate as substrate [83, 86–88].

**Fig. 4:**
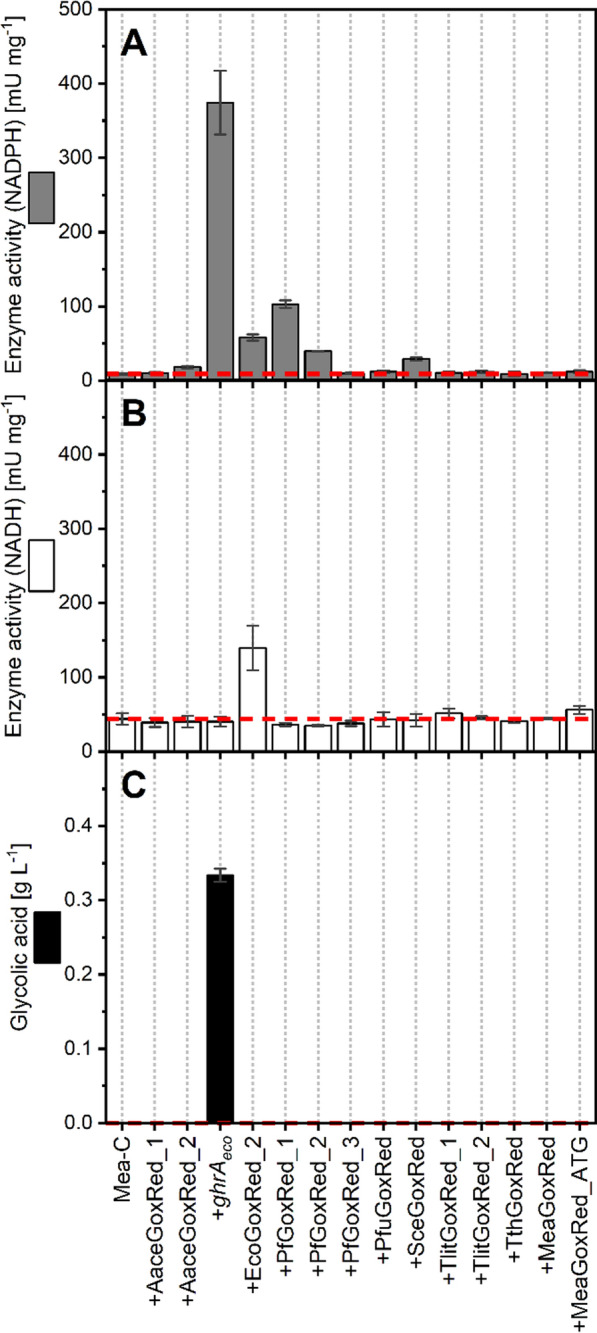
Glyoxylate reductase screening in *M. extorquens* TK 0001. Measured enzyme activities of the expressed glyoxylate reductases during enzyme assay using **A** NADPH and **B** NADH as redox cofactors. **C** Associated GA production in the corresponding shake flask cultures of the engineered strains using methanol minimal medium. The control strain Mea-C was used as reference (dashed red line). Data and standard deviation represent three independent biological replicates (n = 3)

The most active enzyme was GhrA_eco_ supporting a NADPH-dependent glyoxylate reductase activity of 374.34 ± 42.80 mU mg_Protein_^−1^, followed by PfGoxRed_1, EcoGoxRed_2 (GhrB_eco_), PfGoxRed_2, and SceGoxRed (Fig. 4A, Table S3 in Supplemental File S02). In contrast only the expression of *ghrB*_*eco*_ supported significant glyoxylate reductase enzyme activity using NADH as redox cofactor, in particular 139.23 ± 29.78 mU mg_Protein_^−1^ (Fig. 4B). The latter observation is in line with the literature reporting that GhrA from *E. coli* (*ycdW*, in this study *ghrA*_*eco*_) is a strictly NADPH-dependent glyoxylate reductase, whereas GhrB (*yiaE*, in this study *ghrB*_*eco*_) is a NADH-dependent hydroxypyruvate reductase, which also accepts NADPH [88].

Although successful overexpression is indicated as well for PfuGoxRed, TlitGoxRed_1, TlitGoxRed_2, and MeaGoxRed_ATG by SDS PAGE (Figure S5 in Supplemental File S02), these enzymes showed no measurable activity. In these cases, commonly known factors such as dysfunctional gene expression, enzyme formation (i.e. protein production and folding), or enzyme inactivity due to the specific cytosolic conditions in *M. extorquens* TK 0001 are assumed.

Strikingly, the formation of GA in methanol fermentation was solely observed for the strain *M. extorquens* TK 0001 + pTE1887-*ghrA*_*eco*_ (Mea-GA1) reaching a titer of 0.33 ± 0.01 g_GA_ L^−1^ (Fig. 4C). This production is clearly associated with the increased enzyme activity provided by expression of *ghrA*_*eco*_, exceeding the enzyme activity of the second-best NADPH-dependent enzyme PfGoxRed_1 by 263%. Interestingly, no formation of GA was observed for any other enzyme tested, although substantial enzyme activity was detected, especially for strains overproducing PfGoxRed_1 or NADH-dependent EcoGoxRed_2.

The reason for the lack of GA production despite measurable enzyme activity is yet not understood. The data suggest that a certain threshold of enzyme activity must be exceeded in vivo to successfully divert glyoxylate, a central metabolite of the serine cycle, into GA. This implies that a robust flux is required that overcomes the competing fluxes and regulations that affect the glyoxylate pool of native metabolic pathways (such as the serine cycle and EMCP) [26, 89]. Consequently, this threshold for in vivo enzyme activity in *M. extorquens* TK 0001 is presumably within the range of measured enzyme activities of PfGoxRed_1 or EcoGoxRed_2 (103 mU mg_Protein_^−1^ or 139.23 ± 29.78 mU mg_Protein_^−1^, no GA formation) and GhrA_eco_ (374 mU mg_Protein_^−1^, GA formation observed). Conceivable factors that prevent sufficient enzyme activity in vivo might be unfavorable K_m_ values of the enzymes or regulatory effects that have not been investigated yet.

Interestingly, overproduction of active NADH-dependent EcoGoxRed_2 did not result in detectable product titers, although modeling suggested that NADH-dependent GA production may be slightly favored over NADPH-dependent GA formation (Table 2, see 3.1). In addition to the assumption that the enzyme activity in vivo did not exceed the required threshold, the redox status of the cell may play a role.

In summary, a suitable gene *ghrA*_*eco*_ was identified and its overexpression unlocked GA production in *M. extorquens* TK 0001 by supporting sufficient in vivo GhrA_eco_ enzyme activity. The subsequent work in this study was based on this initial producer strain Mea-GA1.

### Characterization of the initial GA producer strain

**Fig. 5:**
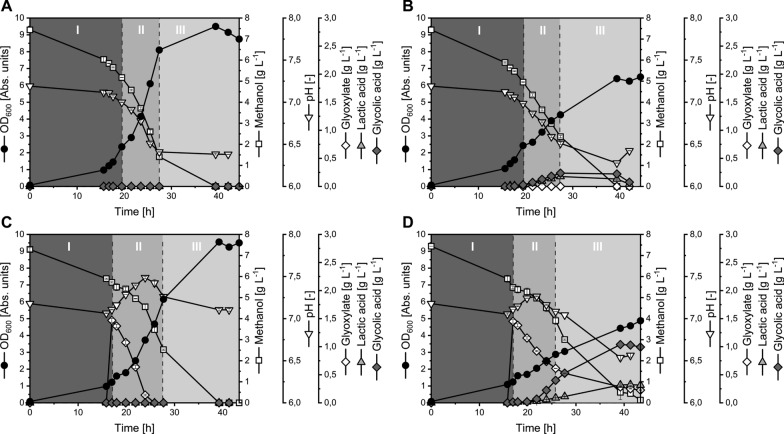
Growth and production behavior of *M. extorquens* TK 0001 strains Mea-C and GA producing Mea-GA1 expressing *ghrA* from *E. coli*. The figures correspond to cultivations using the strains **A** Mea-C, **B** Mea-GA1*,* **C** Mea-C with glyoxylate feeding, **D** Mea-GA1 with glyoxylate feeding*.* Feed of 1.5 g L^−1^ glyoxylate boosts GA production. Growth phases I–III are separated by dashed lines. Data reflect mean values and standard deviation from independent biological duplicates

Subsequently, the initial GA producer strain Mea-GA1 and the control strain Mea-C were characterized under standard cultivation conditions regarding their growth and production behavior (Fig. 5). Compared to the control strain Mea-C, harboring only the empty plasmid, Mea-GA1 showed reduced biomass formation (Fig. 5B), a slower growth rate within the exponential growth phase and only 46.8% of Y_X/MeOH,wild-type_ (Table 4). This physiological change reflects the expected redirection of carbon from biomass to product formation.

The expression of *ghrA*_*eco*_ unlocked the production of GA and initially a titer of 0.24 g_GA_ L^−1^ with a volumetric productivity of 20.17 mg_GA_ L^−1^ h^−1^ (Table 3) was reached. In respect to the corresponding biomass yield (Y_X/MeOH_ of 0.22 g_CDW_ g_MeOH_^−1^ (7.17 g_CDW_ mol_MeOH_^−1^)) and product yield (Y_GA/MeOH_ of 0.08 g_GA_ g_MeOH_^−1^ (33.85 mmol_GA_ mol_MeOH_^−1^); (Table 4)) the performance of the initial producer strain must be ranked in the lower ranges of the in silico calculated yield space.

**Table 3:** Summary of (by-)product titers and volumetric productivities of the initial *M. extorquens* TK 0001 GA producer strain Mea-GA1 and second generation strains Mea-GA2 and Mea-GA3

Strain	GX Feed	GA_max_ [g_GA_ L^−1^]	LA [g_LA_ L^−1^]	Q_GA_ [mg_GA_ L^−1^ h^−1^]	Q_LA_ [mg_LA_ L^−1^ h^−1^]
Mea-GA1	−	0.24	0.18	20.17	15.03
	+	1.04	0.32	44.34	13.64
Mea-GA2	−	0.26	0.30	11.36	13.01
	+	0.53	0.26	21.34	10.41
Mea-GA3	−	0.15	0.18	13.04	15.04
	+	0.64	0.46	23.13	16.73

The maximal glycolic acid (GA_max_) and the corresponding lactic acid (LA) titer are given as mean of independent biological duplicates. Volumetric productivities Q_GA_ and Q_LA_ were determined over the time period from induction until reaching GA_max_. Strains were cultivated without (−) and with supplementation of 1.5 g L^−1^ glyoxylate ( +) to the medium 1 h after induction time point (GX feed)

**Table 4:** Summary of the key performance indicators of *M. extorquens* TK 0001 strains Mea-C and GA producing Mea-GA1 expressing *ghrA* from *E. coli*

Strain	GX Feed	GP	μ [1 h^-1^]	q_MeOH_ [mmol_MeOH_ g_CDW_^−1^ h^−1^]	q_Gox_ [mmol_Gox_ g_CDW_^−1^ h^−1^]	q_C_ [mmol_C_ g_CDW_^−1^ h^−1^]	q_GA_ [mmol_GA_ g_CDW_^−1^ h^−1^]	q_LA_ [mmol_LA_ g_CDW_^−1^ h^−1^]	Y_X/S_ [g_CDW_ mol_C_^−1^]	Y_GA/S_ [mmol_GA_ mol_C_^−1^]	Y_LA/S_ [mmol_LA_ mol_C_^−1^]	Y_GA/X_ [mmol_GA_ g_CDW_^−1^]	Y_LA/X_ [mmol_LA_ g_CDW_^−1^]
Mea-C	−	I	0.16	−16.18	–	−16.18	0.00	0.00	10.06	0.00	0.00	0.00	0.00
		II	0.16	−10.29	–	−10.29	0.00	0.00	15.32	0.00	0.00	0.00	0.00
		III	−0.02	0.00	–	0.00	0.00	0.00	–	–	–	–	–
Mea-GA1		I	0.17	−16.02	–	−16.02	0.12	0.21	10.49	7.56	13.07	0.72	1.25
		II	0.07	−10.17	–	−10.17	0.34	0.21	7.17	33.85	20.44	4.72	2.85
		III	0.03	−2.94	–	−2.94	−0.08	−0.06	8.67	–	–	–	–
Mea-C	+	I	0.16	−16.88	−3.22	−23.33	0.00	0.00	6.96	0.00	0.00	0.00	0.00
		II	0.16	−10.45	−2.51	−15.48	0.00	0.00	10.21	0.00	0.00	0.00	0.00
		III	0.03	−1.91	0.00	−1.91	0.00	0.00	14.90	0.00	0.00	0.00	0.00
Mea-GA1		I	0.17	−7.27	−2.22	−11.70	0.23	0.20	14.11	19.88	17.14	1.41	1.22
		II	0.09	−10.31	−1.77	−13.85	1.21	0.19	6.53	87.68	13.54	13.43	2.07
		III	0.03	−4.42	−0.20	−4.82	0.51	0.14	6.24	105.21	29.14	16.86	4.67
							−0.10	−0.03					

Strains were cultivated under standard conditions without (−) and with ( +) additional supplementation of 1.5 g L^−1^ glyoxylate (GX Feed). Specific substrate uptake rates are given for each methanol (q_MeOH_) and glyoxylate (q_Gox_) individually and as cumulated carbon uptake rate (q_C_). Specific product formation rates are given for GA (q_GA_) and for the main by-product LA (q_LA_). Biomass- and product-substrate yields (Y_X/S_, Y_GA/S_, Y_LA/S_) refer to the consumed molar carbon amount. Product-biomass yields are given for GA (Y_GA/X_) and LA (Y_LA/X_) individually. All performance parameters were determined individually for the three observed growth phase (GP I-III). Data reflect means of independent biological duplicates

Together with the NADPH demand for synthesis of the heterologous enzyme, the GA producer strain Mea-GA1 potentially faces an increased NADPH demand by GhrA_eco_ and forming GA which may compete with the NADPH demand for biomass synthesis and thus reduce biomass yield [88]. The additional expenses of NADPH for GA production could be avoided by examination of NADH-dependent isoforms of the catalyzing enzyme glyoxylate reductase. However, no NADH-dependent glyoxylate reductase which supports GA formation in *M. extorquens* was identified in the screening procedure.

In later process optimization the common trade-off between biomass formation and GA production could be further diminished by applying a two-stage process strategy with initial biomass production followed by a switch to pure GA formation ensuring increased productivity and carbon efficiency [90, 91]. The latter process concept is in reach due to existence of EFMs supporting GA production without biomass formation (Table 2) and known strategies to limit cell growth of this microbe [43, 92].

Another issue that currently limits the efficient GA production is the apparent uptake and assumed utilization of the product towards the end of the fermentation (Fig. 5B). This behavior must clearly be addressed in future developments, for example by identification of corresponding transporters and subsequent strain engineering, which has been recently shown viable for production of dicarboxylic acids with *M. extorquens* AM1 [39].

### Glyoxylate supply is a bottleneck for GA synthesis in *M. extorquens*

Next it was investigated if the supply of the precursor molecule glyoxylate is a bottleneck for GA formation. To do so, cultivations of the strains Mea-GA1 and Mea-C were supplemented with 1.5 g L^−1^ glyoxylate 1 h after induction to evaluate if GA formation is promoted.

First of all, the supplementation of glyoxylate had no negative effect on growth rate or final biomass concentration of the control strain Mea-C (Fig. 5C, Table 4). Moreover, no GA formation was observed for Mea-C, despite providing the precursor compound. The latter suggests that no native glyoxylate reductase is active in the wild-type, which is in line with the obtained enzyme assay results and highlights once more the enabler role of GhrA_eco_ for GA production with *M. extorquens*.

In contrast, the feeding of glyoxylate considerably improved GA formation of Mea-GA1 resulting in a fourfold increase of GA titer to 1.04 g_GA_ L^−1^ (Table 3, Fig. 5D). Thereby, the initial GA producer strain can compete with engineered *S. cerevisiae* harnessing the C_5_-compound xylulose as carbon source and producing 1.0 g L^−1^ GA [44]. However, the titer achieved by Mea-GA1 can yet not compete with that achieved by fermentation of engineered *K. lactis* on ethanol or engineered *E. coli* on glucose reaching 15.0 g_GA_ L^−1^ to up to 65.5 g_GA_ L^−1^, respectively [44].

When supplying glyoxylate, the specific product formation rate q_GA_ of Mea-GA1 of 1.21 mmol_GA_ g_CDW_^−1^ h^−1^ (92 mg_GA_ g_CDW_^−1^ h^−1^) is remarkably high and Y_GA/X_ is nearly tripled. The increased product formation of Mea-GA1 is accompanied with intensified redirection of substrate utilization which leads to a 10% reduction in Y_X/S_ compared to conditions without glyoxylate feeding (Table 4). The supplementation of glyoxylate also improved the product-substrate yield to 0.21 C-mol_GA_ C-mol_MeOH+glyoxylate_^−1^ (Table 4).

These promising results regarding product-substrate yield underline the potential of GA production with *M. extorquens* which can nearly compete with overproducers like engineered *K. lactis* reaching a C-molar yield of 0.32 C-mol_GA_ C-mol_EtOH_^−1^ (recalculated from [44]). However, this comparison pinpoints the important role of a sufficient glyoxylate supply for GA formation in *M. extorquens*.

In conclusion, the addition of glyoxylate boosts GA production substantially in *M. extorquens*, highlighting that glyoxylate supply is indeed a key success factor for high-yield product formation [26]. Additionally, the results prove that the present enzyme activity of GhrA_eco_ in Mea-GA1 does not limit the product formation in vivo. It is more likely that the intracellular glyoxylate supply fueled by the EMCP is a bottleneck. Moreover, the regulation of the intracellular glyoxylate concentration is thought to have an effect on production performance by mediating enzyme activity of GhrA_eco_. Therefore, the next engineering target was to improve glyoxylate regeneration to enhance GA production further. For this purpose, the initial GA producer strain Mea-GA1 was subsequently engineered to overexpress the gene for the ethylmalonyl-CoA mutase (Ecm) which catalyzes a central step within the EMCP [93]. Previously, it was shown that *ecm* overexpression leads to a 2.6-fold increase of the intracellular glyoxylate pool [51], supporting the presupposition of the approach.

### Overexpression of *ecm* in *M. extorquens* has minor effect on GA formation

To enhance glyoxylate regeneration, the initial GA producer strain Mea-GA1 was engineered to overexpress the native *ecm* or the *ecm* from *R. sphaeroides* ATCC 17029, respectively.

**Fig. 6:**
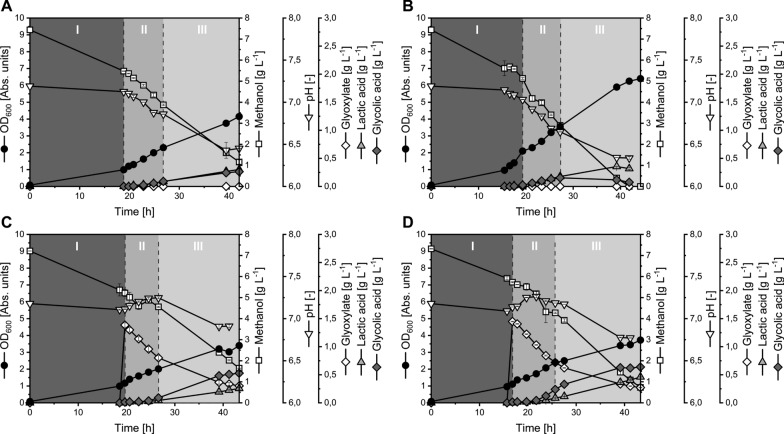
Growth and production behavior of GA producing *M. extorquens* TK 0001 strains Mea-GA2 and Mea-GA3 expressing *ghrA*_*eco*_ from *E. coli* and *ecm*_*mea*_ or *ecm*_*rsh*_ from *M. extorquens* or *R. sphaeroides*, respectively. The figures correspond to cultivations using the strains **A** Mea-GA2, **B** Mea-GA3, **C** Mea-GA2 with glyoxylate feeding, **D** Mea-GA3 with glyoxylate feeding. Supplementation of 1.5 g L^−1^ glyoxylate boosts GA production. Growth phases I–III are indicated by grey boxes with dashed lines. Data reflect mean values and standard deviation from independent biological duplicates

The resulting strains Mea-GA2 (additional copy of *ecm*_*mea*_) and Mea-GA3 (additional copy of *ecm*_*rsh*_) were characterized under standard conditions. During the first growth phase, the growth and production behavior of Mea-GA3 resembled that of Mea-GA1 under the same conditions. Surprisingly, in the second growth phase Mea-GA3 showed a reduction of q_GA_ and Y_GA/MeOH_ by 25–30%. In addition, Y_GA/X_ was reduced by 21% compared to Mea-GA1 (Tables 4, 5). The performance of Mea-GA3 resulted eventually in a reduced GA titer of 0.15 g_GA_ L^−1^ which matches only 62.5% of that achieved by Mea-GA1 (Table 3). Moreover, GA is metabolized by Mea-GA3 even before full depletion of the substrate methanol (Fig. 6B).

**Table 5:** Performance parameters of GA producing *M. extorquens* TK 0001 strains Mea-GA2 and Mea-GA3 expressing *ghrA*_*eco*_ from *E. coli* and *ecm*_*mea*_ or *ecm*_*rsh*_ from *M. extorquens* or *R. sphaeroides*, respectively

Strain	GX Feed	GP	μ [1 h^-1^]	q_MeOH_ [mmol_MeOH_ g_CDW_^−1^ h^−1^]	q_Gox_ [mmol_Gox_ g_CDW_^−1^ h^−1^]	q_C_ [mmol_C_ g_CDW_^−1^ h^−1^]	q_GA_ [mmol_GA_ g_CDW_^−1^ h^−1^]	q_LA_ [mmol_LA_ g_CDW_^−1^ h^−1^]	Y_X/S_ [g_CDW_ mol_C_^−1^]	Y_GA/S_ [mmol_GA_ mol_C_^−1^]	Y_LA/S_ [mmol_LA_ mol_C_^−1^]	Y_GA/X_ [mmol_GA_ g_CDW_^−1^]	Y_LA/X_ [mmol_LA_ g_CDW_^−1^]
Mea-GA2	−	I	0.13	−14.83	–	−14.83	0.15	0.29	9.08	10.34	19.54	1.14	2.15
		II	0.11	−12.44	–	−12.44	0.31	0.21	8.54	24.78	16.96	2.90	1.99
		III	0.04	−5.36	–	−5.36	0.14	0.15	6.82	26.93	28.10	3.95	4.12
Mea-GA3		I	0.16	−16.38	–	−16.38	0.13	0.23	9.96	7.96	14.11	0.80	1.42
		II	0.07	−10.42	–	−10.42	0.25	0.25	6.48	24.26	23.81	3.74	3.67
		III	0.04	−3.88	–	−3.88	−0.04	0.11	9.30	–	29.54	–	3.18
			–	–	–	–	–	−0.08	–	–	–	–	–
Mea-GA2	+	I	0.14	−16.82	−3.08	−22.99	0.29	0.25	5.97	12.64	10.77	2.12	1.80
		II	0.06	−4.54	−2.16	−8.86	0.31	0.09	6.91	34.63	10.41	5.01	1.51
		III	0.03	−6.14	−0.46	−7.05	0.39	0.16	4.64	55.26	22.85	11.91	4.93
Mea-GA3		I	0.16	−9.68	−1.54	−12.76	0.29	0.23	12.39	22.87	18.08	1.85	1.46
		II	0.08	−11.09	−1.95	−14.99	0.84	0.22	5.50	55.73	14.44	10.14	2.63
		III	0.03	−6.33	−0.30	−6.93	0.26	0.24	3.69	37.17	35.26	10.07	9.55

Strains were cultivated under standard conditions without (−) and with ( +) additional supply of 1.5 g L^−1^ glyoxylate (GX Feed). Specific substrate uptake rates are given for the substrates methanol (q_MeOH_) and glyoxylate (q_Gox_) individually and as cumulated carbon uptake rate (q_C_). Specific product formation rates are given for GA (q_GA_) and for the main by-product LA (q_LA_). Biomass- and product-substrate yields (Y_X/S_, Y_GA/S_, Y_LA/S_) refer to the consumed molar carbon amount. Product-biomass yields are given for GA (Y_GA/X_) and LA (Y_LA/S_) individually. All performance parameters were determined individually for the three observed growth phases (GP I-III). Data reflect means of independent biological duplicates

In contrast, Mea-GA2 reached an 8% higher titer, although productivity (Q_GA_) reached only about 56% compared to Mea-GA1 (Table 3). This contradiction can be probably explained by the elevated q_GA_ of 0.14–0.15 mmol_GA_ g_CDW_^−1^ h^−1^ of Mea-GA2 during the first and last growth phase. In combination with the notably increased biomass yield (11% more than Mea-GA1 in growth phase II), the increased specific production rate led to the higher titer of 0.26 g_GA_ L^− 1^ (Tables 3, 5, Fig. 6A).

Despite the data suggesting a successful overproduction of an active Ecm enzyme, growth and production of strains Mea-GA2 and Mea-GA3 seems to be only marginally affected. Nevertheless, a cultivation was conducted with supplementation of 1.5 g L^−1^ glyoxylate (Fig. 6C, D). Again, these conditions enhanced GA titers produced by Mea-GA2 and Mea-GA3 considerably by factor 2 and 4. Compared to conditions without glyoxylate supply, Q_GA_ increased as well by 88% and 77%, respectively. However, production performance of Mea-GA1 was yet not outperformed by Mea-GA2 and Mea-GA3.

In consequence, the results suggest that the intracellular glyoxylate pool was not sufficiently enhanced by the overexpression of *ecm* homologues to boost GA production*.* These observations are in contrast to previously published results that demonstrated an increase of the glyoxylate pool by *ecm* overexpression [51]. The only sign indicating an increased availability of glyoxylate is the increased Y_X/S_ of Mea-GA2 compared to Mea-GA1 which may enhance growth capabilities of the strain despite glyoxylate consumption due to GA production. However, it has to be examined in more detail if *ecm* overexpression really affects the growth performance of *M. extorquens* under GA production conditions.

Future strain development should focus on enhancing the glyoxylate regeneration by the EMCP. The enzymes of this pathway, including the Ecm used in this study, exhibit strong cofactor dependencies. For example, cobalt ion-containing vitamin B_12_ [40, 94] and limited cobalt concentration was shown to induce a metabolic bottleneck that might consequently limit glyoxylate regeneration [40, 95]. Thus, as shown before [95], providing higher cobalt (and/or vitamin B_12_) concentration might abolish the metabolic limitation of strains Mea-GA2 and Mea-GA3 and lead to increased intracellular glyoxylate levels as demonstrated by Cui et al*.* [51].

Apart from the role of cofactors, it is reasonable to assume that a feedback inhibition acts on Ecm, similar to that shown for key branch points in other bacteria [96, 97] considering its assumed role as control point within the EMCP [51]. In this case, mutagenesis could be performed on Ecm to alleviate this inhibition as shown for example for arginine mediated feedback inhibition of ArgB of *C. glutamicum* [98, 99].

Another conceivable effect currently limiting glyoxylate regeneration might be unfavourable expression levels resulting from the polycistronic expression of the *ghrA*_*eco*_ and *ecm* genes from a multi-copy plasmid under control of a strong promoter [29] in Mea-GA2 and Mea-GA3. Fine-tuned expression of individual genes was shown to entail great potential for improving the production performance of microbes such as *C. glutamicum* or *E. coli* [100, 101]. Therefore, applying previously developed promoter libraries, comprising various constitutive and inducible promotors of distinct strength [28, 29], is of great interest for balancing *ecm* overexpression in *M. extorquens* TK 0001.

Finally, as discussed, improving GA production of engineered *M. extorquens* is not as straightforward as simply overexpressing *ecm*. It rather seems inevitable that multiple, coordinated metabolic interventions of the EMCP, the serine cycle, and most likely on regulatory level are probably required to improve product formation beyond the performance of Mea-GA1 [51].

### Lactic acid occurs as a by-product

In order to validate the observed GA production, fermentation samples were derivatized to derive silyl esters and analyzed using GC–MS measurement and presence of GA was confirmed. Strikingly, lactic acid (LA) was found to be a major by-product formed by all GA producing strains, namely Mea-GA1, Mea-GA2 and Mea-GA3 (Figure S6 in Supplemental File S02).

The LA titer formed by Mea-GA1 amounted with 0.18 g_LA_ L^−1^ to about 75% of the obtained GA titer (Table 3). In contrast, Mea-GA2 and Mea-GA3, overexpressing *ecm*, produced even 15–20% more LA than GA under standard conditions, which was unforeseen since the *ecm* overexpression targeted to increase glyoxylate availability and thereby GA production (Table 3). Interestingly, additional glyoxylate supplementation boosted GA formation of all three strains to a larger extent than LA formation, so that the LA titer was only 30 – 70% of the GA titer (i.e. 1.04 g_GA_ L^−1^ vs. 0.30 g_LA_ L^−1^ for Mea-GA1 and 0.64 g_GA_ L^−1^ vs. 0.46 g_LA_ L^−1^ for Mea-GA3) (Table 3). Besides, the other KPIs showed no discernable trend between GA and LA formation (Tables 4, 5). Taken together the obtained results indicate a potential connection between the EMCP and LA formation as well as between C_2_ (GA formation) and C_3_ metabolism in *M. extorquens*. Possible mechanisms that lead to the observed coupled GA and LA synthesis will be discussed in more detail in “Analysis of coupled GA and LA production by *M. extorquens*” section.

### Fermentative production of a GA/LA mixture in lab scale bioreactors

Finally, a fed-batch process with the best performing strain Mea-GA1 was conducted to potentially improve the production by applying substrate feeding and controlled cultivation conditions.

**Fig. 7:**
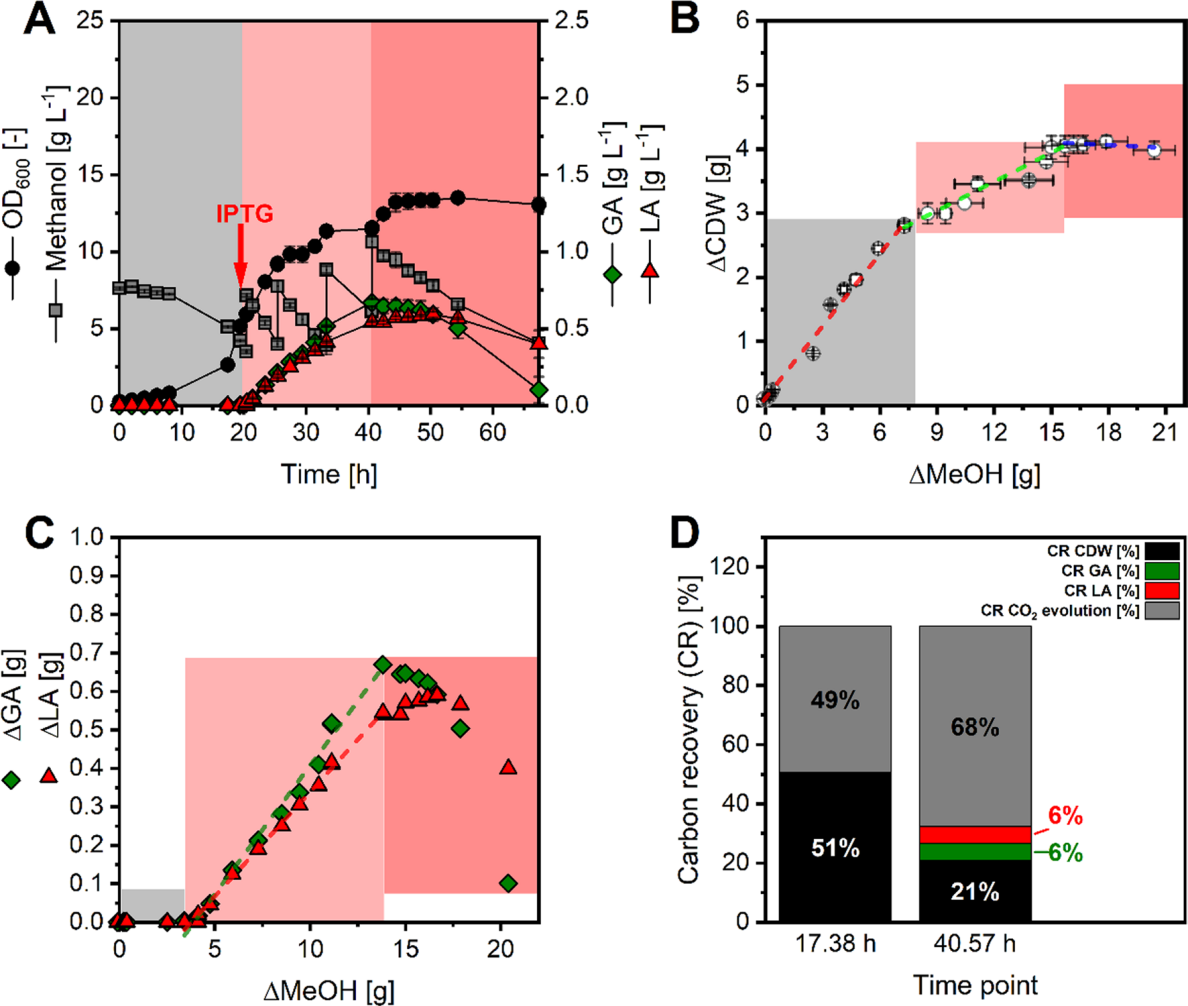
Growth and production behavior of GA producing Mea-GA1 in fed-batch fermentation. Shown is **A** the time-dependent development of optical density measured at 600 nm (black circles), methanol concentration (grey squares), GA concentration (green diamonds), and LA concentration (red triangle). **B** Development of biomass-substrate yield Y_X/MeOH_ during the three growth phases. The slopes of the linear fits represent Y_X/MeOH_ in the three phases I (red dashed line), II (green dashed line), and III (blue dashed line). **C** Development of product-substrate yield Y_P/S_ during the three growth phases for GA (green diamonds), and LA (red triangles). The slopes of the linear fits represent Y_P/S_ for GA (green dashed line) and LA (red dashed line). Growth phases are indicated by colored boxes: I (grey), II (rose), III (light red). **D** Carbon recovery (CR) of measured products in relation to utilized methanol. The time points represent the end of batch phase prior to IPTG induction (17.38 h) the end of production phase (40.57 h). Carbon recovery includes the proportion of carbon captured in biomass (estimated from average biomass composition of bacteria [105]) and CO_2_ evolution, which is an estimated value (leading to 100% carbon recovery) since CO_2_ release was not measured during the fermentation. Data reflect mean values and deviation from independent biological duplicates

During the batch phase (Growth phase I), the non-induced strain showed a short lag phase of about 8 h, grew exponentially with a µ_max_ of 0.15 ± 0.00 h^−1^ and achieved a Y_X/MeOH_ of 0.37 ± 0.02 g_CDW_ g_MeOH_^−1^. Methanol was rapidly consumed after the lag phase with a specific substrate uptake rate q_S_ of 0.41 ± 0.02 g_MeOH_ g_CDW_^−1^ h^−1^. (Fig. 7A, B) These values match the range of published data for the related *M. extorquens* AM1 strain [26].

When OD_600_ reached a value of 5, gene expression of glyoxylate reductase was induced by addition of a final concentration of 1 mM IPTG. Interestingly, growth slowed down to a µ_max_ of 0.08 ± 0.00 h^−1^ (Growth phase II). Additionally, Y_X/MeOH_ was substantially reduced to 0.15 ± 0.01 g_CDW_ g_MeOH_^−1^. However, carbon uptake was increased by 26% compared to the batch phase and remained at a high level (0.53 ± 0.03 g_MeOH_ g_CDW_^−1^ h^−1^) (Fig. 7A, B).

Subsequently to IPTG induction, steady formation of GA and LA was observed. GA was formed with a Y_GA/MeOH_ of 67.0 ± 1.8 mg_GA_ g_MeOH_^−1^ (28.20 ± 0.76 mmol_GA_ mol_MeOH_^−1^) (Fig. 7C) and a specific production rate q_p_ of 35.5 ± 0.9 mg_GA_ g_CDW_^−1^ h^−1^ (0.467 mmol_GA_ g_CDW_^−1^ h^−1^). A final titer of 0.67 g_GA_ L^−1^ was reached after 41 h. Interestingly, LA formation in growth phase II followed a similar pattern as GA formation (Fig. 7A). Specifically, a Y_LA/MeOH_ of 54 ± 0.9 mg_LA_ g_MeOH_^−1^ (19.20 ± 0.32 mmol_LA_ mol_MeOH_^−1^) and a q_P_ of 28.6 ± 0.9 mg_LA_ g_CDW_^−1^ h^−1^ (0.317 mmol_LA_ g_CDW_^−1^ h^−1^) was obtained (Fig. 7C). The titer of LA was with 0.54 g_LA_ L^−1^ slightly lower compared to GA after 41 h. Finally, the volumetric productivities of GA and LA in phase II were 37 mg_GA_ L^−1^ h^−1^ and 27 mg_LA_ L^−1^ h^−1^, respectively.

Recently, high cell densities (OD_600_ ~ 100) were achieved in bioreactor cultivation of a state-of-the-art synthetic methylotrophic *E. coli* strain grown on methanol. This strain was further engineered to produce several products from methanol and reached for LA a titer of 284 mg_LA_ L^−1^, a carbon yield of 52.2 C-mmol_LA_ mol_MeOH_^−1^, and a volumetric productivity of 12.7 mg_LA_ L^−1^ h^−1^ [102]. Notably, our engineered native methylotroph Mea-GA1 delivered for the individual products GA and LA titers, rates and yields that are either in the same order of magnitude or even significantly higher—a fact, that was not to be expected, given that production of chemicals with *M. extorquens* usually results in very low titers, rates, and yields [4, 25].

The successful upscaling of the bioprocess to a stirred bioreactor delivered deeper information about the production kinetics, while the KPI showed a minor improvement compared to previous experiments. During the production phase, the growth rate and biomass yield decrease and specific productivities are low despite a high carbon uptake. These observations point towards an increased dissimilation of methanol. A carbon recovery analysis (Fig. 7D) was performed to analyze the fate of carbon in the two phases. It indicates an estimated 19% higher net CO_2_ release during the production phase compared to growth phase. This is assumed to occur due to increased dissimilation via formate into CO_2_ counterbalancing some elevated energy (ATP) demands.

Moreover, the fermentation data (Table 6) showed a remarkable structural coupling between GA and LA production, as the number of carbon atoms entering both products, was almost identical 56.38 ± 1.51 C-mmol_GA_ C-mol_MeOH_^−1^ for GA and 57.55 ± 0.96 C-mmol_LA_ C-mol_MeOH_^−1^ for LA as was the specific production rates in C-molar metrics for GA (0.93 ± 0.02 C-mmol_GA_ g_CDW_^−1^ h^−1^) and LA (0.95 ± 0.03 C-mmol_LA_ g_CDW_^−1^ h^−1^) formation. The latter results emphasize the above suggested coupling of GA and LA formation at the metabolic level that is yet not understood.

**Table 6:** Key performance indicators of GA producing Mea-GA1 in fed-batch fermentation

GP	Strain	μ [1 h^-1^]	q_MeOH_ [C-mmol g_CDW_^−1^ h^−1^]	q_GA_ [C-mmol g_CDW_^−1^ h^−1^]	q_LA_ [C-mmol g_CDW_^−1^ h^−1^]	Y_X/MeOH_ [g_CDW_ mol_C_^−1^]	Y_GA/MeOH_ [C-mmol mol_C_^−1^]	Y_LA/MeOH_ [C-mmol mol_C_^−1^]
I	Mea-GA1	0.15 ± 0.00	12.70 ± 0.56	0	0	11.85 ± 0.64	0	0
II		0.08 ± 0.00	16.54 ± 0.94	0.93 ± 0.02	0.95 + 0.03	4.81 ± 0.32	56.38 ± 1.51	57.55 ± 0.96

The performance parameters are derived individually for the batch phase (Growth phase I, GP I) and production phase (Growth phase II, GP II). Specific product formation rates are given for GA (q_GA_) and for the main by-product LA (q_LA_). Biomass- and product-substrate yields (Y_X/S_, Y_GA/S_, Y_LA/S_) refer to the consumed molar carbon amount of methanol

After 40 h of fermentation, the growth rate decreased further and cells entered stationary phase while the carbon uptake remained high. This was specifically the case because product formation ceased and the products were taken up again and converted alongside with methanol. To date, the fate of the carbon of either GA, LA or MeOH in this phase remains unclear since no biomass was formed and no other products (except CO_2_, which was not measured) were detected by using the established analytical methods (data not shown). One explanation might be an increased respiration to account for increased ATP demand (coupled with CO_2_ formation) or the burden of overproduction of the glyoxylate reductase. In addition, it is known that several bacterial strains, and also methylotrophic strains like *M. extorquens*, produce Polyhydroxyalkanoates as a carbon storage product under nutrient limitation [103] reflecting another carbon sink. Such limitations, also of cofactors of key enzymes (see e. g. vitamin B_12_ dependent EMCP enzymes [104]), have to be considered in further process development.

A recent study for *M. extorquens* AM1 demonstrates a strategy to cope with the observed uptake of GA. Pöschel et al. (2022) observed that their producer strain of mesaconic acid and 2-methylsuccinic acid was taking up both products when the cells entered stationary phase. By knocking out the corresponding transporter genes, the uptake of 2-methylsuccinic acid was significantly reduced and mesaconic acid uptake was completely diminished [39]. Examining the role of these transporters, especially the identified DctA transporter, for GA and LA product uptake and identifying specific GA and LA transporters is of great interest for future strain development.

### Analysis of coupled GA and LA production by *M. extorquens*

In this section we further discuss possible mechanisms that lead to the observed coupling of GA and LA synthesis. In *M. extorquens*, native lactate dehydrogenase (LDH) catalyzes the one-step reaction forming LA from pyruvate and NADH [36, 81]. In addition, a potential LA forming pathway exists that uses methylglyoxal (see “Metabolic modeling” section). The elevated LA production by the initial GA producer strain Mea-GA1 indicates that the organism faces an increased pyruvate/methylglyoxal or/and NADH pool and that LDH or the methylglyoxal pathway functions as valve to balance these pools. Pyruvate is considered as metabolic setpoint whose intracellular concentration remains remarkably constant upon changing metabolic states (growth on succinic acid and methanol) [106]. Also, it is not known if the methylglyoxal pathway is really present in vivo in *M. extorquens*. We therefore hypothesize that an increased availability or surplus of NADH causes formation of LA.

To shed light on the metabolic state of GA and LA co-producing *M. extorquens*, EFMA using the core model was applied. To constrain the model and adapt it to the experimental results of the enzyme assay, only the NADPH-dependent glyoxylate reductase was used for EFMA.

**Fig. 8:**
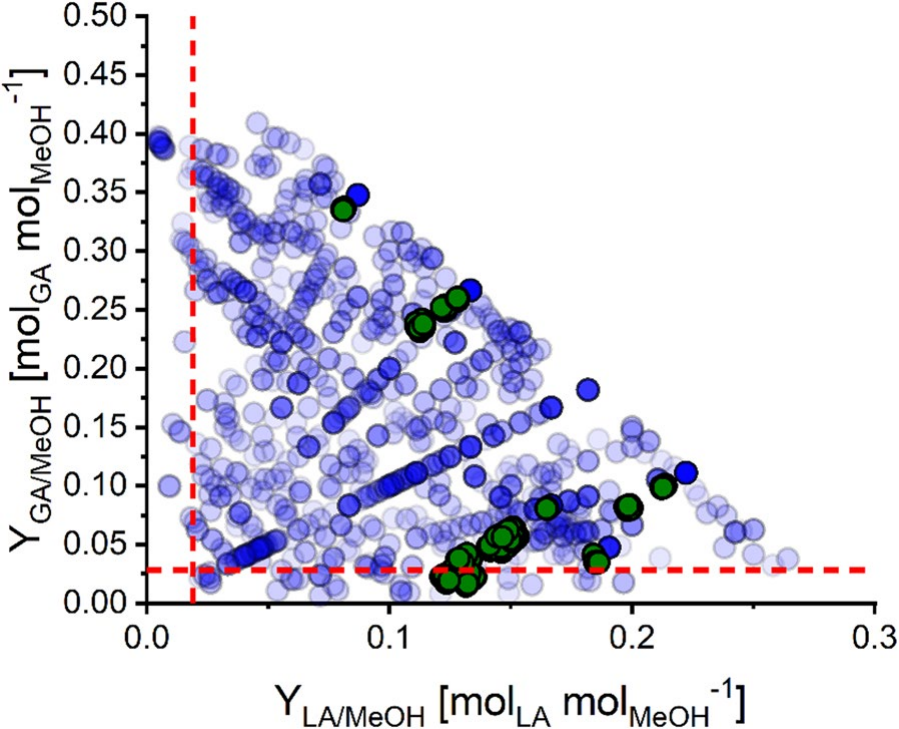
Simulated GA-LA yield-space formed by EFMs which support GA and LA production either with growth (green filled circles) or without growth (blue filled circles) of the *Mextorquens* CoreModel. The red reference lines depict measured Y_GA/MeOH_ and Y_LA/MeOH_ in growth phase II of Mea-GA1 during the fed-batch fermentation. No EFM exists at the intersection of the reference lines indicating that no single EFM exists in the model that can (alone) resolve the metabolic state of the network for the measured Y_P/S_, hence, a combination of EFMs is likely to occur

Interestingly, a smaller fraction of modes (7760) supported co-production of GA and LA of which some even allow growth-coupled co-production (Fig. 8, green filled circles, 5952). None of the growth-coupled modes do closely match the experimentally determined yields of the fermentation (for GA: 28.2 mmol_GA_ mol_MeOH_^−1^, for LA: 19.2 mmol_LA_ mol_MeOH_^−1^; see the dashed red lines in Fig. 8 that represent the measured yields of the fermentation) indicating that the flux distribution in the cell is a combination of certain elementary flux modes.

To get an idea why the observed GA-LA coupling occurs, the EFMs were analyzed in more detail. As a first result we found that the methylglyoxal pathway does not occur in GA and LA producing growth-coupled EFMs. We therefore exclude this pathway as a source of the observed LA. Next, contribution of the redox state of the cell was analyzed. It was found that the transhydrogenase reaction (R087) occurred only in 181 (all non-growing) EFMs forming GA and LA, while the NADPH demand was mainly fulfilled by oxidative PPP (present in 5321 EFMs). An active transhydrogenase would allow for independent conversion of surplus NADH into NADPH. However, genes encoding for transhydrogenases are repressed in methanol-grown *M. extorquens* AM1 [106] assuming transhydrogenase inactivity as well for the used *M. extorquens* TK001. These results suggest that an excess of NADH is available when GA is synthesized enforcing the formation of LA by lactate dehydrogenase.

A deletion of lactate dehydrogenase may resolve the GA/LA coupling. In addition, the overproduction of an active transhydrogenase under methylotrophic growth conditions could counterbalance NADH excess and provide NADPH for GA synthesis. Such approaches, improving the redox power for the target pathway, were shown to be viable in particular cases like the 1,4-butanediol study of Genomatica [107].

### Intervention strategies enforcing growth-coupled GA synthesis

As a perspective for future work and motivated by the found EFMs that couple growth with GA production (see “Elementary flux mode analysis: synthesis of GA” section), we used the concept of minimal cut sets (MCSs, [108]) to determine metabolic engineering strategies that would enforce growth-coupled high-yield GA production in *M. extorquens*. Specifically, we searched for MCSs (with a maximum of 8 reaction knockouts) that target all modes producing less than 0.125 mol_GA_ mol_MeOH_^−1^. In addition, at least one mode should remain ensuring a product yield above this threshold with a minimal biomass yield of 0.094 g_CDW_ g_MeOH_^−1^ (only EFMs with NADPH-dependent glyoxylate reductase were considered as this variant was experimentally investigated herein). In total, 48 MCSs were derived with 6 (1 MCS), 7 (7 MCS), and 8 (40 MCS) interventions. The smallest MCS was examined in detail, which suggests the following deletions:

1. R002: 2 oxidized-cytochrome-c + 1 fald_p + 1 h2o_c = 3 h_p + 1 for_p + 2 reduced-cytochrome-c
2. R010: 1 mleneh4mpt_c + 1 nad_c = 1 nadh_c + 1 menylh4mpt_c
3. R041: 1 atp_c + 1 pi_c + 1 pyr_c = 1 h_c + 1 pep_c + 1 amp_c + 1 ppi_c
4. R043: 1 coa_c + 1 pyr_c + 1 nad_c = 1 co2_c + 1 nadh_c + 1 accoa_c
5. R083: 2 oxidized-cytochrome-c + 1 h2o_c + 1 pyr_c = 2 h_c + 1 co2_c + 1 ac_c + 2 reduced-cytochrome-c
6. R085: 1 asp__L_c = 1 nh4_c + 1 fum_c

First of all, this MCS suggests deletion of R002 to avoid periplasmic C_1_ oxidation, connected with an efflux of electrons towards oxygen. The deletion of R010 couples the C_1_ assimilation with the generation of NADPH by NADP-dependent methylene-H4MPT dehydrogenase (MtdA) that is important for GA production by NADPH-dependent glyoxylate reductase. The other four predicted deletions are less intuitive but they all protect drain of electrons (e.g. R083) or indirect conversion of NADPH to NADH via different cycles to eventually generate an excess of NADPH that can only be balanced by the (here used NADPH-dependent) synthesis of GA. To prove correctness of the suggested engineering strategy, growth-coupled GA production was tested by performing parsimonious FBA (maximization of growth rate) under application of the six reaction knockouts (flux set to zero). The simulation showed that growth-coupling indeed worked and that 0.217 mol_GA_ mol_MeOH_^−1^ were formed at the maximal growth rate in this mutant, which was calculated to be 0.154 h^−1^. The flux distribution of this scenario is shown in Figure S4 in Supplemental File S02. Genetic implementation of this extensive intervention strategy goes beyond the scope of this work but it highlights stoichiometric constraints of high-yield GA production and might serve as a promising starting point for future metabolic engineering.

## Conclusions

The fermentative production of GA from methanol by the engineered *M. extorquens* strains of this study is still a long way to reach titers that are considered sufficient for commercialization of a biotechnological process [6]. The strain Mea-GA1 produced GA with a final yield of 0.067 g_GA_ g_MeOH_^−1^ representing 5.6% of the theoretical maximal yield. Interestingly, lactic acid was formed as a by-product with up to 0.054 g_LA_ g_MeOH_^−1^. Nevertheless, the performance metrics obtained in this study, as well as the important and attractive role of methanol as a future alternative carbon source [4, 25], indicate a promising starting point for the development of a methanol-based process for the production of GA or of LA using *M. extorquens*. In comparison to state-of-the-art synthetic methylotrophs the engineered strain showed competitive performance [102]. Moreover, to date we are not aware of methanol-based production of GA using any microbes, although (synthetic) methylotrophic LA production was demonstrated e.g. using *Ogataea polymorpha*, *Pichia pastoris* or *E. coli* [102, 109, 110]. Compared to photosynthetic microorganisms that convert CO_2_ directly to glycolate our strain *M. extorquens* GA1 achieved a higher volumetric productivity. However, photoautotrophic processes using *Chlamydomonas reinhardtii* [111–115] reach high titers with steady production (up to 4.5 g_GA_ L^−1^) and show carbon yields of up to 82% [112, 114]. Consequently, further work is required to strengthen the competitiveness of our microbial cell factory.

Our modeling results suggest that the periplasmic route of C_1_ assimilation, which is less efficient in terms of ATP yield compared to the cytoplasmic route, is likely to be active and contributing to the carbon intake of *M. extorquens* due to excellent fit of modeled and in vivo growth rate measurements. But, so far, no studies are available in literature that quantify the contribution of this pathway. As a consequence, it remains puzzling to what extent cytoplasmic and periplasmic C_1_ assimilation cooperate in the examined strain.

Metabolic modeling of methanol-based GA formation of *M. extorquens* revealed that the entire carbon of the substrate can be conserved in the product. We also found a possible metabolic engineering strategy that enforces growth-coupled GA overproduction by generating an excess of NADPH. The genetic implementation of this extensive intervention strategy was out of scope of this work but could be a starting point for future work. Questions remain regarding the mechanisms and the exact underlying metabolism that result in LA formation. We believe that a surplus of NADH is formed during GA formation which cannot be drained by the native metabolic architecture. In this context, formation of LA acts as a valve for NADH excess since a transhydrogenase seems to be not operational in *M. extorquens* when growing on methanol [106]. While the production of a by-product like LA was not intended herein, the co-production of LA can be advantageous in a commercial sense. In recent years, LA has gained interest as commodity chemical derived from fermentation because of its versatile potential for various applications in food, chemical, cosmetic, and pharmaceutical industries, and also as a building block for PLA [116]. Moreover, LA and GA are used to produce the biodegradable co-polymer PLGA that is applied e. g. for drug delivery systems [44, 117]. The viability of a bio-based production of the co-polymer, especially directly in the cell, has been demonstrated before [118, 119]. Furthermore, the production of GA is biotechnologically established and as well commercialized by the company Metabolic Explorer using glucose as carbon source [47]. Consequently, the methanol-based production of a mixture of GA and LA, or the individual products, might be as well of great interest for commercial applications. The present study makes a step forward delivering know-how, strains and engineering targets for methanol-based production of C_2_ and C_3_ products. The application of model-based techniques such as minimal cut sets may help identify targets to enhance the performance of native methylotrophic microbial cell factories for the synthesis of GA, LA, or other products.

## Supplementary Information

Supplementary material 1.

Supplementary material 2.

## Data Availability

The presented *Mextorquens* CoreModel supporting the conclusions of this article is freely available in the *Mextorquens* CoreModel repository, accessible under https://github.com/JoFa-IGB/MextorquensCoreModel.git.
