# Supplementary material for: A novel engineered strain of Methylorubrum extorquens for methylotrophic production of glycolic acid
Source: Microb Cell Fact. 2024 Dec 23;23:344. doi: 10.1186/s12934-024-02583-y (PMC11665112; doi:10.1186/s12934-024-02583-y)
Supplement: Supplementary file 2 — Supplementary material 2. [file 12934_2024_2583_MOESM2_ESM.docx]

A novel engineered strain of *Methylorubrum extorquens* for methylotrophic production of glycolic acid

**Supplemental File #2**

Katharina Dietz^1,#^, Carina Sagstetter^1,#^, Melanie Speck^1,#^, Arne Roth^1^, Steffen Klamt^2^ and Jonathan Thomas Fabarius^1,*^

^1^Fraunhofer Institute for Interfacial Engineering and Biotechnology, Straubing branch BioCat, Schulgasse 11a, Straubing, Germany

^2^Max Planck Institute for Dynamics of Complex Technical Systems, Sandtorstr. 1, Magdeburg, Germany

*Correspondence: jonathan.fabarius@igb.fraunhofer.de (J. T. Fabarius)

^#^Authors contributed equally

**Table S1. List of used primers in this study.** All primer sequences used to clone the indicated codon-optimized Glyoxylate reductase genes into the NcoI restriction site of pTE1887 or the Ethylmalonyl-CoA mutase genes ecm_mea_ and ecm_rsh_ into pTE1887-ghrA_eco_ using the BamHI restriction site.

| **Primer name** | **Sequence 5' 🡪 3'** | **T_m_ [°C]** |
| --- | --- | --- |
| fw_*aace1*_pTE1887 | CGACTCTAGAATTAAAGAGGAGAAATTAACCATGTCGTTCCCGCGCGAG | 67 |
| rev_*aace1*_pTE1887 | CGATCTTTTCGAACTGCGGATGGCTCCAGCTCGCCATGTCAGTAGCCGCGCTCCG | 66 |
| fw_*aace2*_pTE1887 | CGACTCTAGAATTAAAGAGGAGAAATTAACCATGAAGCGCCTCGCCATCTC | 67 |
| rev_*aace2*_pTE1887 | CGATCTTTTCGAACTGCGGATGGCTCCAGCTCGCCATGTCAGTAGCCGCGCTCCG | 66 |
| fw_*aace3*_pTE1887 | CGACTCTAGAATTAAAGAGGAGAAATTAACCATGTCGCTCTCGCGCACC | 67 |
| rev_*aace3*_pTE1887 | CGATCTTTTCGAACTGCGGATGGCTCCAGCTCGCCATGTCAGGCCTCGACCGGCG | 68 |
| fw_*ghrA_eco_*_pTE1887 | CGACTCTAGAATTAAAGAGGAGAAATTAACCATGGACATCATCTTCTACCACCCG | 65 |
| rev_*ghrA_eco_*_pTE1887 | CGATCTTTTCGAACTGCGGATGGCTCCAGCTCGCCATGTCAGTAGCCGCGGGCGC | 69 |
| fw_*ghrB_eco_*_pTE1887 | CGACTCTAGAATTAAAGAGGAGAAATTAACCATGAAGCCGTCGGTCATCCTG | 66 |
| rev_*ghrB_ec_*_o__pTE1887 | CGATCTTTTCGAACTGCGGATGGCTCCAGCTCGCCATGTCAGTCGGCGACGTGCG | 67 |
| fw_*pf1*_pTE1887 | CGACTCTAGAATTAAAGAGGAGAAATTAACCATGCCGATGAAGAAGACCGTCC | 66 |
| rev_*pf1*_pTE1887 | CGATCTTTTCGAACTGCGGATGGCTCCAGCTCGCCATGTCAGCCGCGCCAGACC | 67 |
| fw_*pf2*_pTE1887 | CGACTCTAGAATTAAAGAGGAGAAATTAACCATGAAGAAGACCATCCTCGCC | 63 |
| rev_*pf2*_pTE1887 | GATCTTTTCGAACTGCGGATGGCTCCAGCTCGCCATGTCACTTCTTGAAGTGGACGAGG | 63 |
| fw_*pf3*_pTE1887 | CGACTCTAGAATTAAAGAGGAGAAATTAACCATGAACACCGTCGCCCTCATG | 67 |
| rev_*pf3*_pTE1887 | CGATCTTTTCGAACTGCGGATGGCTCCAGCTCGCCATGTCAGTAGCCGAGGAGGCGATC | 67 |
| fw_*pfu*_pTE1887 | CGACTCTAGAATTAAAGAGGAGAAATTAACCATGAAGCCGAAGGTGTTCATC | 62 |
| rev_*pfu*_pTE1887 | CGATCTTTTCGAACTGCGGATGGCTCCAGCTCGCCATGTCACTGCTCGTTGAAGCC | 62 |
| fw_*sce*_pTE1887 | CGACTCTAGAATTAAAGAGGAGAAATTAACCATGTCGAAGAAGCCGATCGTGC | 67 |
| rev_*sce*_pTE1887 | CGATCTTTTCGAACTGCGGATGGCTCCAGCTCGCCATGTCAGACGAGCGGCTTCGAC | 65 |
| fw_*tlit1*_pTE1887 | CGACTCTAGAATTAAAGAGGAGAAATTAACCATGAAGCCGAAGGTGTTCATCACC | 67 |
| rev_*tlit1*_pTE1887 | CGATCTTTTCGAACTGCGGATGGCTCCAGCTCGCCATGTCACGGCGGCGACGAG | 66 |
| fw_*tlit2*_pTE1887 | CGACTCTAGAATTAAAGAGGAGAAATTAACCATGAAGCCGAAGGTCCTCGTG | 66 |
| rev_*tlit2*_pTE1887 | GATCTTTTCGAACTGCGGATGGCTCCAGCTCGCCATGTCAGAGGAGCTTCACCTCCTCG | 67 |
| fw_*tth*_pTE1887 | GCGACTCTAGAATTAAAGAGGAGAAATTAACCATGCGCCCGACCGGC | 67 |
| rev_*tth*_pTE1887 | CGATCTTTTCGAACTGCGGATGGCTCCAGCTCGCCATGTCAGACGACCGGGTTCGGC | 68 |
| fw_*mae*_TTG_pTE1887 | CGACTCTAGAATTAAAGAGGAGAAATTAACCTTGAAGCGCAAGCCGCTGG | 68 |
| rev_*mae* pTE1887 | CTTTTCGAACTGCGGATGGCTCCAGCTCGCCATGTCAGAGCATGCTCGGAAGGATCC | 68 |
| fw_*mae*_ATG_pTE1887 | CGACTCTAGAATTAAAGAGGAGAAATTAACCATGAAGCGCAAGCCGCTGG | 68 |
| fw_*ecmmea*_pTE1887 | GATCGAAGGCCGCCATATGTCGGGCGAAAGAGGAGAAATTAACCATGAGCGCGCAAGCGAG | 65 |
| rv_*ecmmea*_pTE1887 | GCTTACGGTACCTGCAGGACTAGTTCAGGATCTCAGAAGACCTGCGCCC | 65 |
| fw_*ecmrsh*_pTE1887 | ATCGAAGGCCGCCATATGTCGGGCGAAAGAGGAGAAATTAACCATGACCCAGAAGGACTCGCC | 65 |
| rv_*ecmrsh*_pTE1887 | GCTTACGGTACCTGCAGGACTAGTTCAGGATCTCACTCGGCGGCGAGG | 65 |
| 2698 (Colony PCR forward primer) | CCACCTGACGTCTAAGAAACC | 57 |
| 2430 (Colony PCR reverse primer) | GCTAGCTTGGATTCTCACCA | 57 |

**Table S2. Codon-optimized Glyoxylate reductase genes*.*** The following microorganisms were used as donor strains: *Escherichia coli* K12 MG1655 (*ghrA_eco_* and *EcoGoxRed_2*, KEGG entry: b1033 and b3553), *Pseudomonas fluorescens* Pf0-1 (*PfGoxRed_1-3*, KEGG entry: Pfl01_0936, Pfl01_2771, and Pfl01_3899), *Thermococcus litoralis* (*TlitGoxRed_1-2*, KEGG entry: OCC_02245 and OCC_08355), *Pyrococcus furiosus* DSM 3638 (*PfuGoxRed*, KEGG entry: PF0319), *Saccharomyces cerevisiae* (*SceGoxRed*, KEGG entry: YNL274C), *Thermus thermophilus* HB27 (*TthGoxRed*, KEGG entry: TT_C0431), *Acetobacter aceti* (*AaceGoxRed_1-2*, KEGG entry: A0U92_03200, A0U92_11415), and *Methylorubrum extorquens* TK 0001 (*MeaGoxRed*, KEGG entry: TK0001_6029) was cloned starting with ATG instead of the native start-codon TTG, namely *MeaGoxRed_ATG*.

| **KEGG Entry** | **Codon-optimized DNA sequence 5’ 🡪 3’** |
| --- | --- |
| b1033 | ATGGACATCATCTTCTACCACCCGACCTTCGACACCCAGTGGTGGATCGAGGCCCTGCGCAAGGCCATCCCGCAGGCCCGCGTCCGCGCCTGGAAGTCGGGCGACAACGACTCGGCCGACTACGCCCTGGTCTGGCACCCGCCGGTCGAGATGCTCGCCGGCCGCGACCTCAAGGCCGTGTTCGCCCTCGGCGCCGGCGTCGACTCGATCCTCTCGAAGCTGCAGGCCCACCCGGAGATGCTCAACCCGTCGGTCCCGCTGTTCCGCCTGGAGGACACCGGCATGGGCGAGCAGATGCAGGAGTACGCCGTGTCGCAGGTCCTCCACTGGTTCCGCCGCTTCGACGACTACCGCATCCAGCAGAACTCGTCGCACTGGCAGCCGCTGCCGGAGTACCACCGCGAGGACTTCACCATCGGCATCCTGGGCGCCGGCGTGCTCGGCTCGAAGGTCGCCCAGTCGCTCCAGACCTGGCGCTTCCCGCTCCGCTGCTGGTCGCGCACCCGCAAGTCGTGGCCGGGCGTCCAGTCGTTCGCCGGCCGCGAGGAGCTCTCGGCCTTCCTCTCGCAGTGCCGCGTCCTGATCAACCTCCTCCCGAACACCCCGGAGACCGTGGGCATCATCAACCAGCAGCTGCTCGAAAAGCTGCCGGACGGCGCCTACCTGCTCAACCTGGCCCGCGGCGTCCACGTGGTGGAGGATGACCTGCTGGCCGCCCTCGACTCGGGCAAGGTGAAGGGCGCCATGCTCGACGTGTTCAACCGCGAGCCGCTGCCGCCGGAGTCGCCGCTCTGGCAGCATCCGCGCGTGACCATCACCCCGCACGTGGCCGCCATCACCCGCCCGGCCGAGGCCGTCGAGTACATCTCGCGCACCATCGCCCAGCTGGAGAAGGGCGAGCGCGTCTGCGGCCAGGTCGACCGCGCCCGCGGCTACTGACTCGAG |
| b3553 | ATGAAGCCGTCGGTCATCCTGTACAAGGCCCTCCCGGACGACCTGCTGCAGCGCCTCCAGGAGCACTTCACCGTCCACCAGGTGGCCAACCTCTCGCCGCAGACCGTGGAGCAGAACGCCGCCATCTTCGCCGAGGCCGAGGGCCTCCTGGGCTCGAACGAGAACGTCAACGCCGCCCTCCTGGAGAAGATGCCGAAGCTGCGCGCCACCTCGACCATCTCGGTCGGCTACGACAACTTCGACGTCGACGCCCTGACCGCCCGCAAGATCCTGCTCATGCACACCCCGACCGTCCTCACCGAGACCGTGGCCGACACCCTCATGGCCCTGGTCCTCTCGACCGCCCGCCGCGTCGTCGAGGTCGCCGAGCGCGTCAAGGCCGGCGAGTGGACCGCCTCGATCGGCCCGGATTGGTACGGCACCGACGTGCACCACAAGACCCTCGGCATCGTCGGCATGGGCCGCATCGGCATGGCCCTCGCCCAGCGCGCCCACTTCGGCTTCAACATGCCGATCCTCTACAACGCCCGCCGCCACCACAAGGAGGCCGAGGAGCGCTTCAACGCCCGCTACTGCGACCTGGACACCCTGCTGCAGGAGTCGGACTTCGTCTGCCTCATCCTCCCGCTGACCGACGAGACCCACCACCTGTTCGGCGCCGAGCAGTTCGCCAAGATGAAGTCGTCGGCCATCTTCATCAACGCCGGCCGCGGCCCGGTCGTGGATGAGAACGCCCTCATCGCCGCCCTGCAGAAGGGCGAGATCCACGCCGCCGGCCTCGACGTCTTCGAGCAGGAGCCGCTGTCGGTGGACTCGCCGCTGCTGTCGATGGCCAACGTCGTGGCCGTGCCGCACATCGGCTCGGCCACCCATGAGACCCGCTACGGCATGGCCGCCTGCGCCGTCGATAACCTCATCGACGCCCTGCAGGGCAAGGTCGAGAAGAACTGCGTCAACCCGCACGTCGCCGACTGACTCGAG |
| Pfl01_0936 | ATGCCGATGAAGAAGACCGTCCTGGCCTTCTCGCGCATCACCCCGCCGATGATCGAGCGCCTCCAGCAGGACTTCGACGTCATCGTGCCGAACCCGAAGAACGGCGACATCAACGCCCAGTTCAACGAGGCCCTGCCGCACGCCCACGGCCTCATCGGCGTCGGCCGCAAGCTGGGCAAGGCCCAGCTGGAGAACGCCGCCAAGCTGGAGGTGGTCTCGTCGGTGTCGGTCGGCTACGACAACTACGACCTCGCCTACTTCAACGAGCGCGGCATCATGCTGACCAACACCCCGGACGTGCTGACCGAGTCGACCGCCGACCTCGCCTTCGCCCTCATCATGTCGTCGGCCCGCCGCGTCGCCGAGCTCGACGCCTGGACCAAGGCCGGCCAGTGGCAGGCCTCGGTCGGCGCCCCGCTCTTCGGCTGCGATGTGCACGGCAAGACCCTGGGCATCGTCGGCATGGGCAACATCGGCGCCGCCGTGGCCCGCCGCGGCCGCTTCGGCTTCAACATGCCGATCATCTACTCGGGCAACTCGCGCAAGACCGAGCTGGAGCAGGAGCTGGGCGCCCAGTTCCGCTCGCTCGACCAGCTGCTCGCCGAGGCCGACTTCGTCTGCCTCGTCGTGCCGCTGTCGGACAAGACCCGCCACCTCATCTCGCACCGCGAGCTGGCCCTGATGAAGCCGGACGCCATCCTGGTCAACATCTCGCGCGGCCCGGTCGTCGACGAGCCGGCCCTCATCGAGGCCCTCCAGAACAACCGCATCCGCGGCGCCGGCCTCGACGTCTACGAGAAGGAGCCGCTCGCCGAGTCGCCGCTGTTCCAGCTCAAGAACGCCGTGACCCTGCCGCACATCGGCTCGGCCACCAACGAGACCCGCGAGGCGATGGCTAACCGCGCCCTCACCAACCTGCGCTCGGCCCTCCTGGGCGAGCGCCCGCAGGACCTCGTCAACCCGCAGGTCTGGCGCGGCTAACTCGAG |
| Pfl01_2771 | ATGAAGAAGACCATCCTCGCCTTCTCGCGCGTGTCGCCGTCGCTCCTGGAGCCGTACAAGGACGACTACGAGGTCATCATCATCTCGCCGGAGCTGGGCGACATGGACGCCCAGTTCGAGGCCGCCATCTCGAACGCCCACGGCCTCATCGGCGGCTCGCGCCGCCTCGGCGAGGCCGAGCTCGCCGCCGCCGTCAACCTCGAAATCGTGTCGTCGATCTCGGTCGGCTACGACAACTACGACCTCGACTACCTGAACCGCCGCGGCATCATGCTCACCAACACCCCGGACGTCCTCAACGAGACCACCGCCGACCTGGCCCTGGCCCTCATCCTCGCCACCGCCCGCCGCATCCCGGAGCTCGACTCGTGGACCAAGGAGGGCAACTGGACCCAGACCATCGACGCCTCGCAGTTCGGCTGCGACGTGCACGGCAAGACCCTGGGCATCATCGGCCTCGGCAAGATCGGCGAGGCCATCGCCCGCCGCGGCCGCTTCGGCTTCGGCATGACCATCCTGTACTCGGGCAACTCGCGCAAGCCGAAGCTCGAACAGGAGCTCGGCGCCCGCTTCGTGCCGCAGGAGGAGCTCCTCTCGGCCTCGGACTTCGTCTGCCCGGTCGTCCCGCTGACCGACGCCACCCGCAACCTCATCGGCCGCAAGGAGCTCGCCCTGATGGGCCCGGAGTCGATCCTGATCAACATCTCGCGCGGCCCGGTCGTCGACCAGGACGCCCTCATCGAGGCCCTCCAGGAGAAGACCATCCGCGCCGCCGGCCTCGACGTGTACGTGAAGGAGCCGCTGACCAACTCGGAGCTGTTCAACCTGCGCAACGTCGTGACCGTCCCGCACATCGGCTCGGCCACCACCGACACCCGCAACGCGATGGCTAAGCGCGCCCTGGAGAACCTCCTGGCCGGCCTCGAAGGCCGCCAGCCGCGCGACCTCGTCCACTTCAAGAAGTAACTCGAG |
| Pfl01_3899 | ATGAACACCGTCGCCCTCATGTCGCGCGACACCCTGCTCCTGAAGCAGCTGCAGGAGGCCTTCGCCCGCCGCGCCCCGCAGCTCTCGGCCGTCCTCGCCGACGATCCGCGCGCCGTCAACGCCCAGATCGCCGCCTGCTGGTTCCCGCTCTCGGACTCGCTGGCCGCCCTCCCGAACCTCCAGGTCATCCACTCGGTCGCCGCCGGCATCGACCACCTCGAACACGACCCGTCGTGCCCGGACCTCCCGGTCTGCCGCGTCGTCGATCCGGGCCATCGCCAGGGCATGACCGAGTACGTCCGCTGGGCCGTGATCCACTTCCACCGCGGCTTCGACCAGGTGCTCGAACAGCAGCGCCAGCAGCACTGGGAGCGCCCGCTCCAGCGCCCGGCCCACGAGTTCCGCATCGGCGTCATGGGCCTCGGCTCGCTCGGCTCGGCCATCGTCCAGGACCTCGCCTCGGCCGGCTACGACGTGCGCGGCTGGGCCCGCTCGTCGAAGAACCTGCCGGGCGTCCAGACCTACGCCGGCATCGATGCCTTCAACCCGTTCCTCGAAGGCGTCGAGCTGCTCATCAACCTCCTCCCGCTCACCCACGAGACCCGCGGCATCCTGAACCGCCAGACCTTCGAGCGCCTGGGCAACGGCGCCGCCCTCGTCAACGTCGGCCGCGGCGGCCACCTCAACATCGACGACCTGCAGCAGGCCCTCGCCCGCGGCAAGCTCCGCGGCGCCCTCCTCGACGTCTTCGAGCAGGAGCCGCTGCCGGCCGACCACCCGCTCTGGAAGACCCCGGGCGTCACCATCACCCCGCACATGGCCTCGGCCGCCTCGCATGACTGCATCGCCGAGCAGATCGCCGAGAACTTCCGCCGCCTGAACGCCGGCGAGCCGCTCCTCAACTCGGCCGATCGCCTCCTCGGCTACTAACTCGAG |
| OCC_02245 | ATGAAGCCGAAGGTGTTCATCACCCGCCAGATCCCGGAGAACGGCATCAAGATGATCGAGAAGTTCTACGAGATCGAGCTCTGGAAGGACCCGAAGGCCCCGCCGCGCGGCGTCCTCCTCGAAAAGGTCCGCGAGGTGGACGCCCTCGTCACCCTCGTGACCGACAAGGTCGACAAGGAGCTCCTCGAAAACGCCCCGAAGCTCAAGATCATCGCCCAGTACGCCGTGGGCTACGACAACATCGACATCGAGGAGGCCACCAAGCGCGGCATCTACGTGACCAACACCCCGGGCGTGCTCACCGACGCCACCGCCGACCTCGCCTTCGCCCTCCTCCTGGCCGTCGCCCGCCGCATCGTCGAGGCCGACGCCTTCGTGCGCTCGGGCGAGTGGAAGAAGTCGGAGGTGGGCTGGCACCCGCTCATGTTCCTCGGCTACGGCCTGAAGGGCAAGACCCTGGGCATCGTGGGCTTCGGCCGCATCGGCCAGGCCCTCGCCAAGCGCGCCAAGGGCTTCGGCATGAAGATCATCTACTACTCGCGCACCCGCAAGCCGGAGGCCGAGGAGGAGATCGGCGCCGAGTACGTGGACTTCGAGACCCTGCTGAAGGAGTCGGACTTCATCTCGCTGCACGTGCCGCTCACCAAGGAGACCTACCACATGATCGGCGAGAAGGAGCTCAAGCTGATGAAGCCGAACGCCATCCTCATCAACACCTCGCGCGGCGCCGTGGTCGACACCAACGCCCTCATCAAGGCCCTGAAGGAGGGCTGGATCGCCGGCGCCGGCCTCGACGTCTTCGAGGAGGAGCCGTACTACAACGAGGAGCTCTTCAAGCTCAAGAACGTCGTGCTCGCCCCGCACATCGGCTCGGCCACCCATGAGGCCCGCGAGGGCATGGCCGAGCTGGTCGCCAAGAACCTGATCGCCTTCGCCAAGGGCGAGATCCCGCCGAACCTGGTCAACAAGGACGTCCTCACCTCGTCGCCGCCGTAACTCGAG |
| OCC_08355 | ATGAAGCCGAAGGTCCTCGTGCTCTTCAACATGAAGTCGGAGCCGCTCGAACTCCTCAAG  CAGTACTGCGACGTCGACGTGCTCGTGTACCCGGAGAAGGAGAAGATCCTCGAAATCATCGGCGAGTACGACGGCCTCATCGTGTCGCCGCTCAACCGCGTCGACCGCGAGATCATCGAGAAGGGCGAGAAGCTCAAGGTCATCTCGACCCACTCGGCCGGCTACGACCACATCGACCTGAAGGCCGCCACCGAGAAGGGCATCTACGTCACCAAGGTCTCGGGCGTCCTGTCGGAGGCCGTCGCCGAGTTCGCCGTCGGCCTCACCATCGCCCTCCTGCGCAAGATCGCCTACTCGGACAAGTTCATGCGCCGCGGCCTGTGGGACTCGCACCGCACCGTCTGGGGCTGGTACAAGCGCGTCGAGACCGTGTACGGCAAGAAGGTCGGCATCCTGGGCATGGGCCCGATCGGCAAGGCCATCGCCCGCCGCATGAAGGCCCTCGGCACCGAGATCTACTACTGGTCGCGCTCGCGCAAGGAGGACATCGAGAAGGAGGTCTCGGCCAAGTGGCTCCCGCTCGAAGAGGTCCTCAAGCAGTCGGACATCGTGATCCTCGCCCTGCCGTCGACCCCGGAGACCTACCACCTGATCAACGAGGAGCGCCTCAAGCTGATGGAGGGCAAGTACCTGATCAACATCGGCCGCGGCTCGCTGGTCGACGAGAAGGCCCTGATCAAGGCCCTGAAGGAGGGCAAGCTGAAGGGCTTCGCCACCGACGTGTACGAGAAGGAGCCGCTCCAGGAGTCGGAGCTGTTCGAGATGGAGTGGGAGACCGTCCTGACCCCGCACCACGCCGGCCTCGCCAAGGAGGCGATGGAAGACATGGGCTTCCAGGCCGTGAACAACCTCCTCTCGATCTTCAAGGGCGAGATCCCGGAGAACCTGGTCAACAAGGAGGTGCTGAAGATCCGCCCGATCGAGGAGGTGAAGCTCCTCTAACTCGAG |
| PF0319 | ATGAAGCCGAAGGTGTTCATCACCCGCGCCATCCCGGAGAACGGCATCAACATGCTGGAGGAGGAGTTCGAGGTGGAGGTCTGGGAGGAGGAGCGCGAGATCCCGCGCGAGAAGCTCCTGGAGAAGGTGAAGGACGTCGACGCCCTGGTGACCATGCTGTCGGAGCGCATCGACCAGGAGGTCTTCGAGAACGCCCCGCGCCTGCGCATCGTCGCCAACTACGCCGTGGGCTACGACAACATCGACGTCGAGGAGGCCACCCGCCGCGGCATCTACGTCACCAACACCCCGGACGTCCTCACCAACGCCACCGCCGACCACGCCTTCGCCCTGCTCCTCGCCACCGCCCGCCATGTCGTCAAGGGCGACAAGTTCGTCCGCTCGGGCGAGTGGAAGCGCAAGGGCATCGCCTGGCACCCGAAGTGGTTCCTCGGCTACGAGCTCTACGGCAAGACCATCGGCATCGTCGGCTTCGGCCGCATCGGCCAGGCCATCGCCCGCCGCGCCAAGGGCTTCAACATGCGCATCCTGTACTACTCGCGCACCCGCAAGTCGCAGGCCGAGAAGGAGCTGGGCGCCGAGTACCGCCCGCTCGAAGAGGTGCTCAAGGAGTCGGACTTCGTGATCCTGGCCGTGCCGCTCACCAAGGAGACCATGTACATGATCAACGAGGAGCGCCTCAAGCTGATGAAGCCGACCGCCATCCTCGTCAACATCGCCCGCGGCAAGGTCGTGGACACCAAGGCCCTGATCAAGGCCCTCAAGGAGGGCTGGATCGCCGGCGCCGGCCTCGACGTCTTCGAGGAGGAGCCGTACTACAACGAGGAGCTCTTCTCGCTCGACAACGTGGTCCTCACCCCGCACATCGGCTCGGCCACCTTCGAGGCCCGCGAGGCGATGGCTGAGCTCGTGGCCCGCAACCTGATCGCCTTCAAGCGCGGCGAGATCCCGCCGACCCTGGTCAACAAGGAGGTGATCAAGATCCGCAAGCCGGGCTTCAACGAGCAGTAACTCGAG |
| YNL274 | ATGTCGAAGAAGCCGATCGTGCTCAAGCTCGGCAAGGACGCCTTCGGCGACCAGGCCTGGGGCGAGCTCGAAAAGATCGCCGACGTCATCACCATCCCGGAGTCGACCACCCGCGAGCAGTTCCTCCGCGAGGTCAAGGACCCGCAGAACAAGCTCTCGCAGGTCCAGGTCATCACCCGCACCGCCCGCTCGGTGAAGAACACCGGCCGCTTCGACGAGGAGCTGGCCCTCGCCCTGCCGTCGTCGGTCGTCGCCGTCTGCCACACCGGCGCCGGCTACGATCAGATCGACGTGGAGCCGTTCAAGAAGCGCCACATCCAGGTCGCCAACGTGCCGGACCTGGTCTCGAACGCCACCGCCGACACCCACGTCTTCCTCCTGCTGGGCGCCCTCCGCAACTTCGGCATCGGCAACCGCCGCCTCATCGAGGGCAACTGGCCGGAGGCCGGCCCGGCCTGCGGCTCGCCGTTCGGCTACGATCCGGAGGGCAAGACCGTCGGCATCCTCGGCCTGGGCCGCATCGGCCGCTGCATCCTCGAACGCCTGAAGCCGTTCGGCTTCGAGAACTTCATCTACCACAACCGCCACCAGCTCCCGTCGGAGGAGGAGCACGGCTGCGAGTACGTGGGCTTCGAGGAGTTCCTGAAGCGCTCGGACATCGTCTCGGTCAACGTGCCGCTGAACCACAACACCCACCACCTGATCAACGCCGAGACCATCGAGAAGATGAAGGACGGCGTGGTGATCGTCAACACCGCCCGCGGCGCCGTCATCGACGAGCAGGCCATGACCGACGCCCTCCGCTCGGGCAAGATCCGCTCGGCCGGCCTCGACGTGTTCGAGTACGAGCCGAAGATCTCGAAGGAGCTGCTGTCGATGTCGCAGGTCCTCGGCCTCCCGCACATGGGCACCCACTCGGTGGAGACCCGCAAGAAGATGGAGGAGCTCGTCGTCGAGAACGCCAAGAACGTCATCCTGACCGGCAAGGTCCTCACCATCGTGCCGGAGCTCCAGAACGAGGACTGGCCGAACGAGTCGAAGCCGCTCGTCTGACTCGAG |
| TT_C0431 | ATGCGCCCGACCGGCGCCCCGCCGTGGCGCTGGCCGTCGTCGCCGTCGCCGGGCCGCAACCCGACCCCGGTCAAGCGCGGCATGAAGGTGTTCGTCACCCGCACCCTCCCGGGCAAGGCCCTCGATCGCCTGCGCGAGCGCGGCCTCGAAGTCGAGGTGCACCGCGGCCTGTTCCTGCCGAAGGCCGAGCTGCTGAAGCGCGTGGAGGGCGCCGTGGGCCTCATCCCGACCGTCGAGGACCGCATCGACGCCGAGGTGATGGACCGCGCCAAGGGCCTCAAGGTGATCGCCTGCTACTCGGTCGGCGTGGACCACGTGGACCTCGAAGCCGCCCGCGAGCGCGGCATCCGCGTCACCCACACCCCGGGCGTCCTCACCGAGGCCACCGCCGACCTCACCCTCGCCCTCCTCCTGGCCGTCGCCCGCCGCGTCGTCGAGGGCGCCGCCTACGCCCGCGACGGCCTCTGGCGCGCCTGGCACCCGGAGCTCCTCCTCGGCCTCGACCTGCAGGGCCTCACCCTCGGCCTCGTCGGCATGGGCCGCATCGGCCAGGCCGTCGCCAAGCGCGCCCTCGCCTTCGGCATGCGCGTCGTCTACCACGCCCGCACCCCGAAGCCGCTGCCGTACCCGTTCCTGTCGCTGGAGGAGCTGCTGAAGGAGGCCGACGTGGTCTCGCTCCACACCCCGCTGACCCCGGAGACCCACCGCCTCCTCAACCGCGAGCGCCTGTTCGCCATGAAGCGCGGCGCCATCCTGATCAACACCGCCCGCGGCGCCCTCGTCGACACCGAGGCCCTCGTCGAGGCCCTCCGCGGCCACCTCTTCGGCGCCGGCCTCGACGTCACCGACCCGGAGCCGCTCCCGCAGGATCACCCGCTCTACCGCCTCCCGAACGCCGTGATCACCCCGCACATCGGCTCGGCCGGCCGCACCACCCGCGAGCGCATGGCCGAGGTCGCCGTCGAGAACCTCCTGGCCGTGCTCGAAGGCCGCGAGCCGCCGAACCCGGTCGTCTGACTCGAG |
| A0U92_03200 | ATGTCGTTCCCGCGCGAGCGCCCGGTGCTGCTCGATCAGATCGGCGGCCCGATCGCCGCCGTGCTCACCGACTACGCCCAGCAGGTGCGCGTGATCGAGGGCAACCGCGACAACGCCGAGCCGTGGCAGACCGCCGGCGCCGACATCCTCCTGACCGGCCCGTCGCCGGCCTGGGCCAAGGCCCCGGTCTCGCAGCCGGCCTCGTGGCGCGACGGCCCGCGCTGGGTCCAGATCGCCTCGGCCGGCATCGACTCGTTCCCGAAGTGGCTCACCGAGGGCCGCATCGTGACCTGCGGCCGCGGCGACGCCGCCACCCCGATCGCCGAGTACGTGATCTCGGCCCTGCTCCACCACGAGCGCCAGGTCGACGCCCTGCACCCGACCACCCCGCAGGAGTGGACCGACGCCATCGCCCCGTTCCGCCAGAACCCGGTGACCGGCACCCTGCACGGCCGCACCCTCGGCCTCGCCGGCTACGGCGCCATCGGCCGCGCCATCGCCTCGCGCGCCCGCGCCTTCGGCATGCGCATCCGCGTCCTCCGCCGCGGCGCCTGGGCCGAGACCGACGAGGGCATCGAGCCGGTGTCGTCGCTGTCGGAGCTCTTCCGCGAGGCCGACCACCTCATCCTCGCCATGCCGCTGACCTCGGAGACCCGCGGCGTCGTCAACGAGGACATCCTCCGCCACGCCAAGGAGGGCCTCCACCTCGTGAACGTCGCCCGCGGCGCCCTCGTCAACCAGGATGCCCTCCTCCGCGCCCTCGACTCGGGCCGCCCGGCCTTCGCCACCCTCGACGTCACCACCCCGGAGCCGCTGCCGGCCGGCCACCCGTTCTACACCCACCCGCGCGTGCGCCTGACCCCGCACATCTCGTGGGTCGGCCCGGACGTGCGCGCCAACCTCGCCCAGCGCATCCGCACCAACCTGTCGCGCTTCCTCCGCAACGAGCCGCTGCTCGACGTCATCGACCCGGAGCGCGGCTACTAACTCGAG |
| A0U92_11415 | ATGAAGCGCCTCGCCATCTCGGCCGACGGCCCGGAGGCCTGGGCCGGCTGGCGCGACGCCTTCGTCCGCGTCGCCCCGGACCTCCCGACCATCTCGTGGTTCGACCCGGCCTTCGACCCGGCGGACGCCGACTACGTCCTGGTCTGGGAGCCGGCCACCGGCGAGATGACCCGCATGGCCGGCATGCGCGCCATCCTGTCGACCGGCGCCGGCGTCAACCACCTCGTGAACCGCCCGGACTTCCCGGCCCACGTGCCGCTCGTCCGCATGGGCGGCGATCAGACCGCCGTGCTGATGGCCGACTACGTGATGTGGGCCACCATCTCGCTCCTGCGCGACGCCAAGAACTGGACCCGCCAGCAGGAGCAGCACGTCTGGAACCGCAACCCGGTCTTCCGCAACTCGGCCGAGACCCGCGTGGGCATCCTCGGCTTCGGCAACCTGGGCGCCGCCGTCGCCCGCCGCCTCGCCGCCGCCGGCTTCATCGTCTCGGCCTGGTGCCGCTCGGCCCGCGAGGAGAAGCACATCCCGCTGTTCTTCGGCGAGGAGGGCCTGCCGGACTTCCTCTCGTCGGTGGACATCCTCGTCAACCTGCTGCCGTCGACCCCGCGCACCCGCCACATCATCGACGCCGAGCTGCTCTCGCGCCTCAAGCCGGGCGCCGGCTTCATCAACGTGGGCCGCGGCGACCACGTGATCGAGGAGGACCTGCTCATGGCCCTCGACTCGCGCCGCCTGTCGGGCGCCGTCCTCGACGTCGTCGCCACCGAGCCGCTCCCGGCCGACTCGCCGCTCTGGGACCATCCGCGCATCACCCTGACCCCGCACATGGCCTCGGAGGCCTCGCGCGATGCCCAGGCCGCCTACGTCGCCCAGGCCATCAAGGAGCTCGAAGAGGGCTCGACCCCGTCGCTGCTGTACCGCCCGGAGCGCGGCTACTAACTCGAG |
| TK0001_6029 | ATGTCGTCGTTGAAGCGCAAGCCGCTGGTGGTCGTCACGCGGCGTCTGCCGGACGCCGTCGAGACGCGCATGCGCGAATTGTTCGATACCCGCCTCAACCACGATGATGCGCCGCTCTCGCAGGAGGCGCTCTCGGCCGCGATTCGCGAAGCCGACGTGCTCGTGCCCACCGTGACCGACGAGATCGGGGCCGGATTGCTCGCGCAGGCGGGGCCGAACCTGCGGCTCATCGCCAATTTCGGCAACGGCGTCGATCACATCGACGTCGCCGGAGCGCTGGAGCGCGGCATCACGGTCACCAACACGCCCGGCGTGCTGACCGAGGACACCGCCGACATGACCATGGCGCTGATCCTGGCGGTCGCCCGCCGTCTCGCGGAAGGTGCGCGCATCATCCCCGACGACGATTGGACCACGGGATGGTCGCCGACCTGGATGCTCGGCCGCCGCATCACGGGCAAGCGCCTCGGCATCGTCGGCATGGGCCGCATCGGCCAGGCGCTCGCCCGCCGCGCCAAGGCTTTCGGTCTGTCGATCCACTACCACAACCGCCGCCGCGTGCCCTCGCATATCGAGGAATCGCTCGATGCGACCTACTGGGAATCCCTCGACCAGATGCTGGCCCGGGTCGATATCGTCTCGGTCAACTGCCCGCACACGCCCGCGACCTATCACCTGCTCTCGGCCCGCCGCCTGAAGCTGCTCAAGCCCGAGGCGATCGTCGTCAACACCGCCCGCGGCGAGGTGATCGACGAGAACGCGCTCGCGCGCCTGATCGAGGGGGGCGAGATCTCGGCCGCCGGCCTCGACGTGTTCGAGCAGGAGCCGGCGGTGAGCCCGCGTCTCGTGCGGCTCGCCCGCACCGGCAAGGTCGTGCTGCTGCCGCATATGGGCTCGGCGACGCATGAGAGCCGCACCGACATGGGCGAGAAGGTCATCATCAACATCAAGACCTTCATGGATGGCCACCGTCCGCCGGACCGGATCCTTCCGAGCATGCTCTGA |

**Table S3. Enzyme activities of glyoxylate reductases obtained in the enzyme assay screening.**

| **Enzyme** | **Activity [mU mg_Protein_^-1^]** |
| --- | --- |
| GhrA_eco_ (NADPH) | 374.34 ± 42.80 |
| PfGoxRed_1 (NADPH) | 103.05 ± 4.86 |
| EcoGoxRed_2 (NADPH) | 58.00 ± 4.28 |
| EcoGoxRed_2 (NADH) | 139.23 ± 29.78 |
| PfGoxRed_2 (NADPH) | 39.60 ± 0.82 |
| SceGoxRed_2 (NADPH) | 29.21 ± 2.08 |


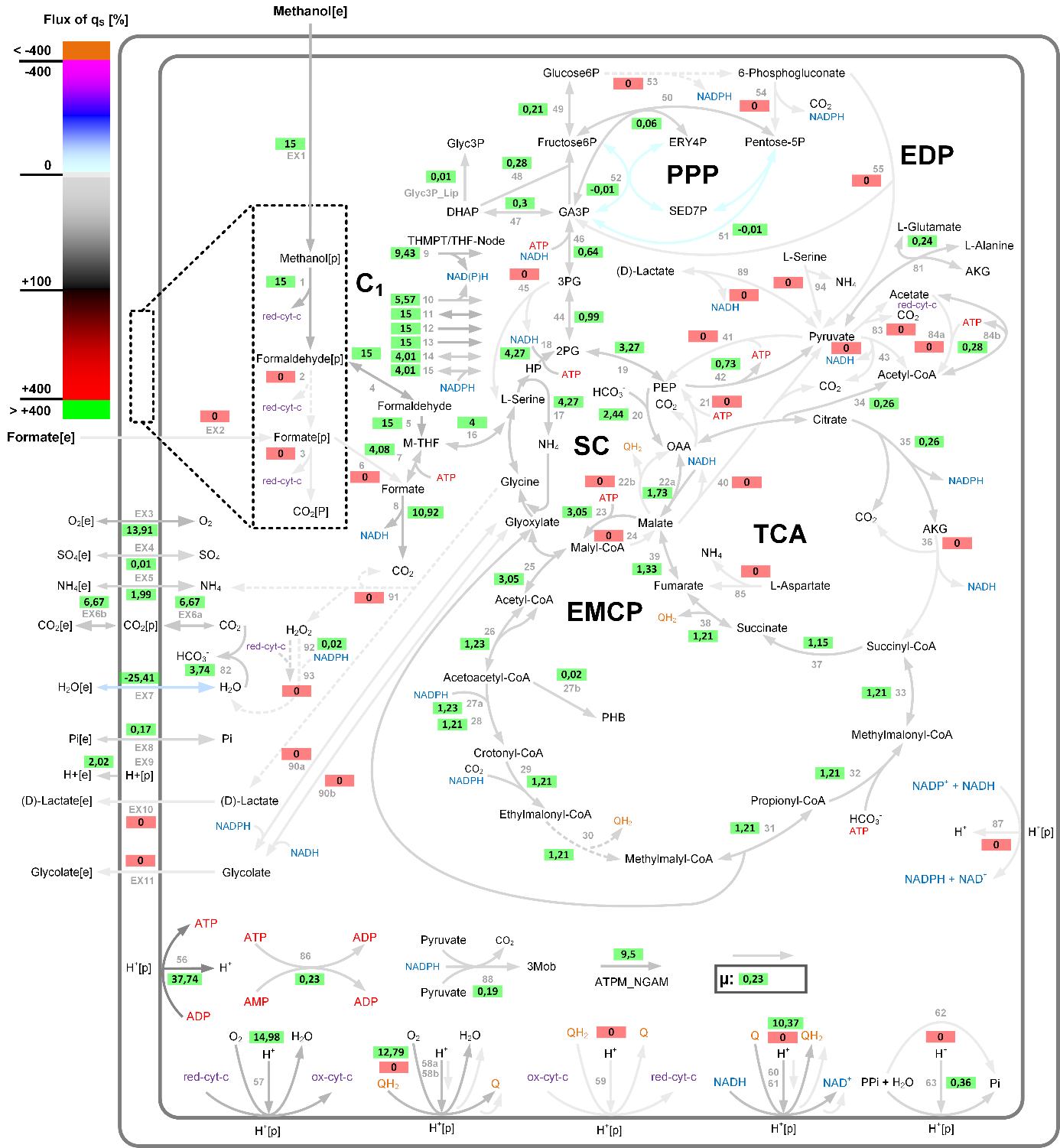


**Figure S1. Map of the central carbon metabolism model of M. extorquens displaying a flux distribution calculated by pFBA with maximal growth rate of 0.23 h^-1^.** Shown are exchange of metabolites, methanol and formate uptake and oxidation, C1-interconversions (THMPT/THF-node), Serine Cycle (SC), Citric Acid Cycle (TCA), Ethylmalonyl-CoA Pathway (EMCP), Pentose Phosphate Pathway (PPP), Entner-Doudoroff Pathway (EDP), respiratory processes, transhydrogenase, ATP generation, growth rate µ, non-growth-associated ATP demand (ATPM_NGAM), and a compressed methylglyoxal pathway for lactic acid formation. The gradual arrow color indicates the fluxes in % relative to methanol uptake (see figure legend). Green boxes indicate active reactions (and their flux value, in mmol g_CDW_^-1^ h^-1^) and red boxes inactive reactions. A summary of abbreviations of metabolites is given in supplement file S01.


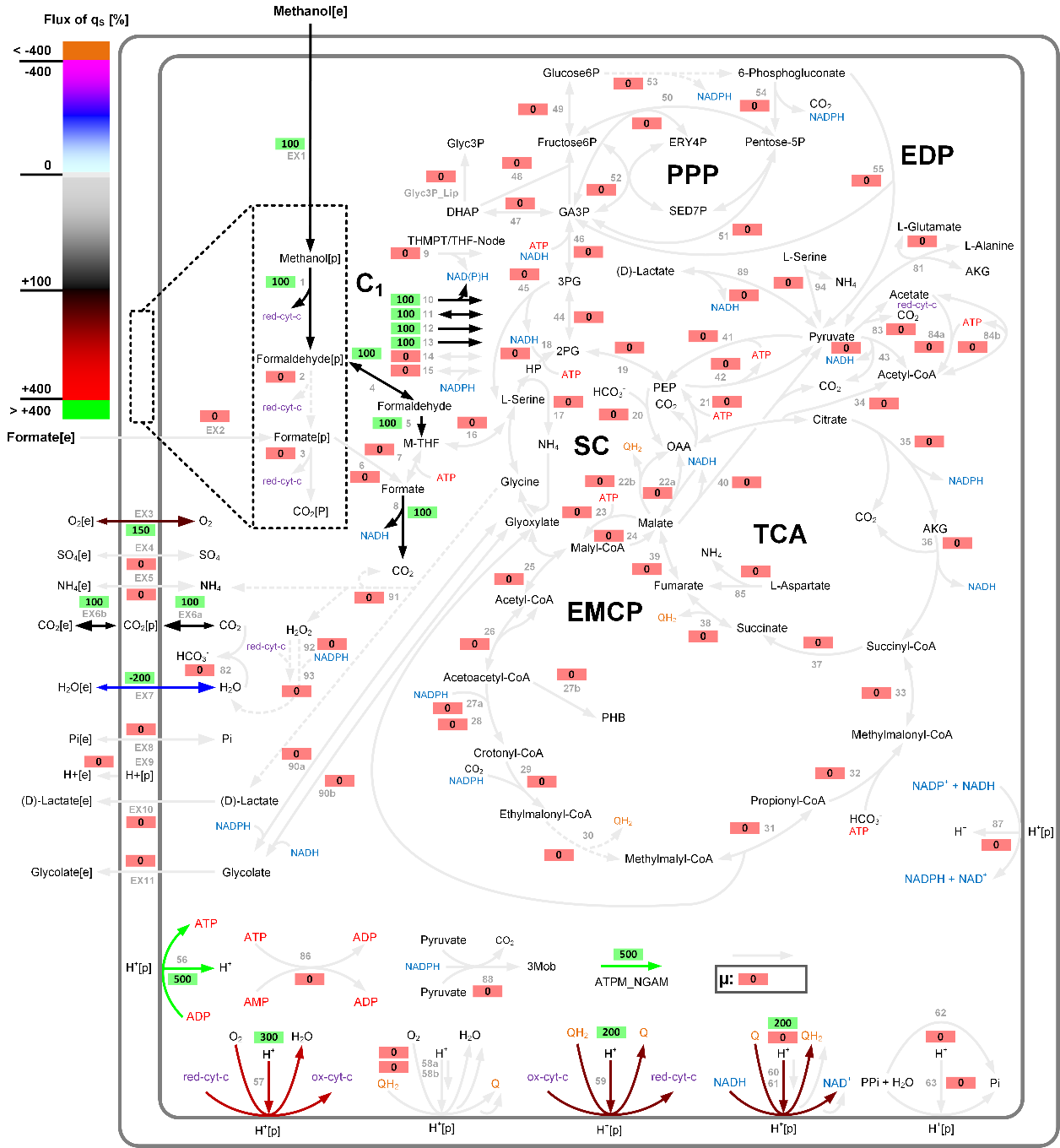


**Figure S2. Map of the central carbon metabolism model of M. extorquens displaying an EFM with maximal ATP yield of 5 mol_ATP_ mol_MeOH_^-1^.** Shown are exchange of metabolites, methanol and formate uptake and oxidation, C1-interconversions (THMPT/THF-node), Serine Cycle (SC), Citric Acid Cycle (TCA), Ethylmalonyl-CoA Pathway (EMCP), Pentose Phosphate Pathway (PPP), Entner-Doudoroff Pathway (EDP), respiratory processes, transhydrogenase, ATP generation, growth rate µ, non-growth-associated ATP demand (ATPM_NGAM), and a compressed methylglyoxal pathway for lactic acid formation. The gradual arrow color indicates the fluxes in % relative to methanol uptake (see figure legend). Green boxes indicate active reactions (and their flux value, % relative to methanol uptake) and red boxes inactive reactions. A summary of abbreviations of metabolites is given in supplement file S01.

**Figure S3. Phase plane with growth rate µ (R_Growth) and oxygen consumption rate q_O2_ (EX003) in the *MextorquensCore* network model.** A strict aerobic growth behavior is predicted by the phase plane.


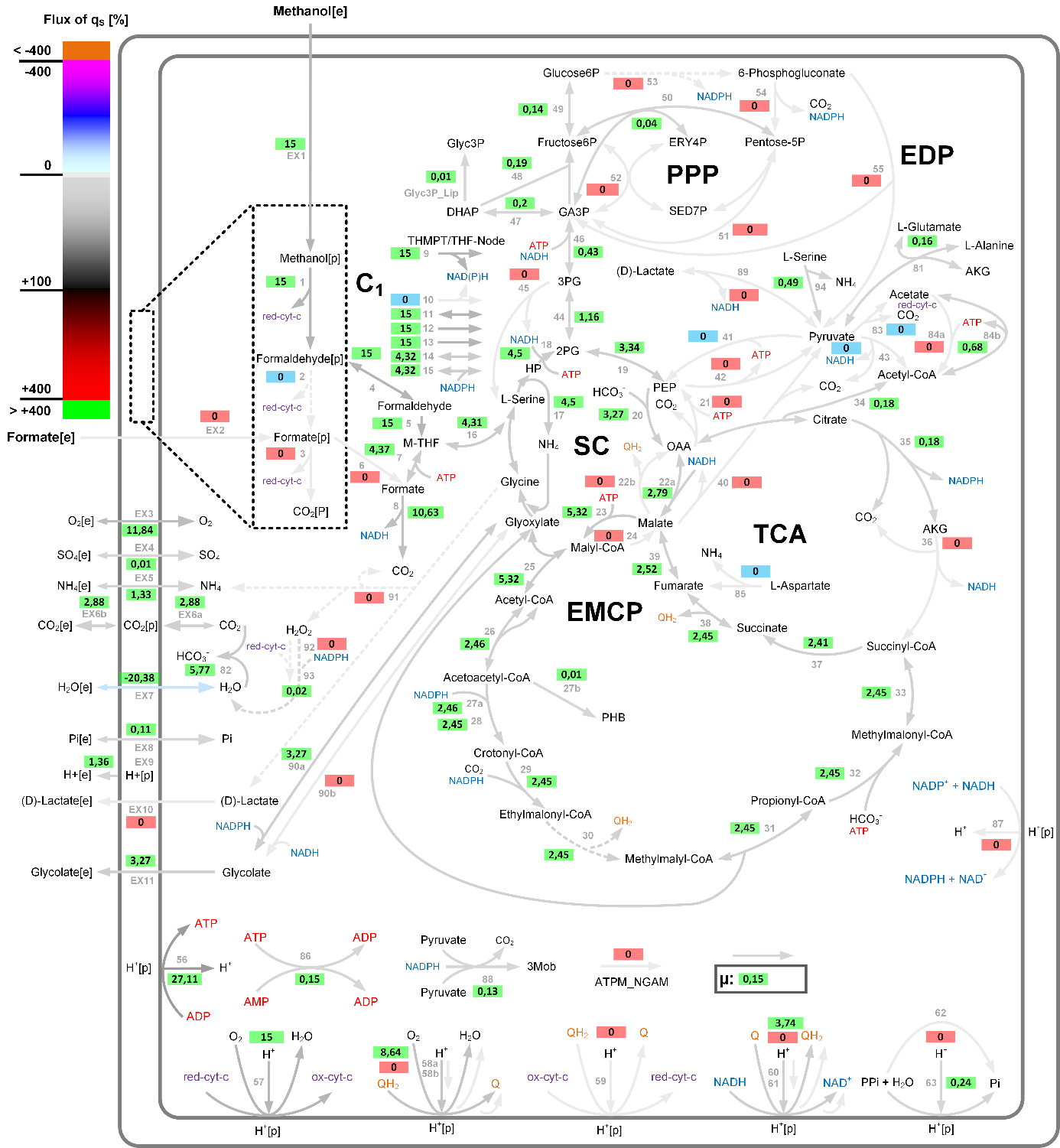


**Figure S4. Map of the central carbon metabolism model of M. extorquens displaying a flux distribution enforced by a MCS with six genetic interventions**. Shown are exchange of metabolites, methanol and formate uptake and oxidation, C1-interconversions (THMPT/THF-node), Serine Cycle (SC), Citric Acid Cycle (TCA), Ethylmalonyl-CoA Pathway (EMCP), Pentose Phosphate Pathway (PPP), Entner-Doudoroff Pathway (EDP), respiratory processes, transhydrogenase, ATP generation, growth rate µ, non-growth-associated ATP demand (ATPM_NGAM), and a compressed methylglyoxal pathway for lactic acid formation. The gradual arrow color indicates the fluxes in % relative to methanol uptake (see figure legend). Green boxes indicate active reactions (and their flux value, in mmol g_CDW_^-1^ h^-1^) and red boxes inactive reactions. The blue boxes indicate the reaction deletions targeted by the MCS to enforce growth-coupled GA production. A summary of abbreviations of metabolites is given in supplement file S01.


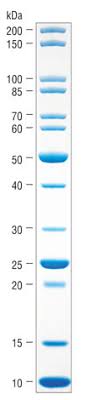

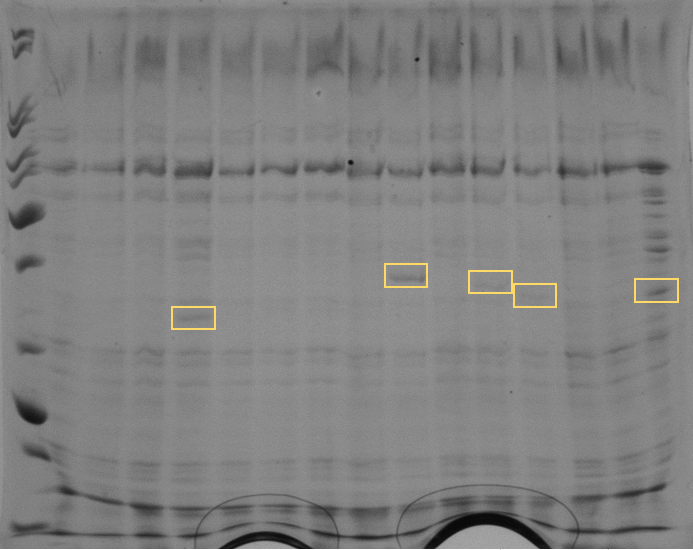


1

2

3

4

5

6

7

8

91

10

11

12

13

14

15

16

**Figure S5. SDS PAGE of overexpressing various glyoxylate reductases in M. extorquens TK 0001 under standard cultivation conditions with given protein sizes.** Lane 1: Unstained Protein Standard P7717, Lane 2: TK 0001 + pTE1887 (Mea-C), Lane3: TK 0001 + pTE1887-aaceGoxRed_1 (36 kDa), Lane 4: TK 0001 + pTE1887-aaceGoxRed_2 (34.5 kDa), Lane 5: TK 0001 + pTE1887-ghrA_eco_ (35.3 kDa), Lane 6: TK 0001 + pTE1887-EcoGoxRed_2 (35.4 kDa), Lane 7: TK 0001 + pTE1887-pfGoxRed_1 (35.6 kDa), Lane 8: TK 0001 + pTE1887-pfGoxRed_2 (35 kDa), Lane 9: TK 0001 + pTE1887-pfGoxRed_3 (34 kDa), Lane 10: TK 0001 + pTE1887-pfuGoxRed (38.3 kDa), Lane 11: TK 0001 + pTE1887-sceGoxRed (38.8 kDa), Lane 12: TK 0001 + pTE1887-tlitGoxRed_1 (36.8 kDa), Lane 13: TK 0001 + pTE1887-tlitGoxRed_2 (37.9 kDa), Lane 14: TK 0001 + pTE1887-tthGoxRed_opt (37 kDa), Lane 15: TK 0001 + pTE1887-meaGoxRed_TTG (36.3 kDa), Lane 16: TK 0001 + pTE1887-meaGoxRed_ATG (36.3 kDa).


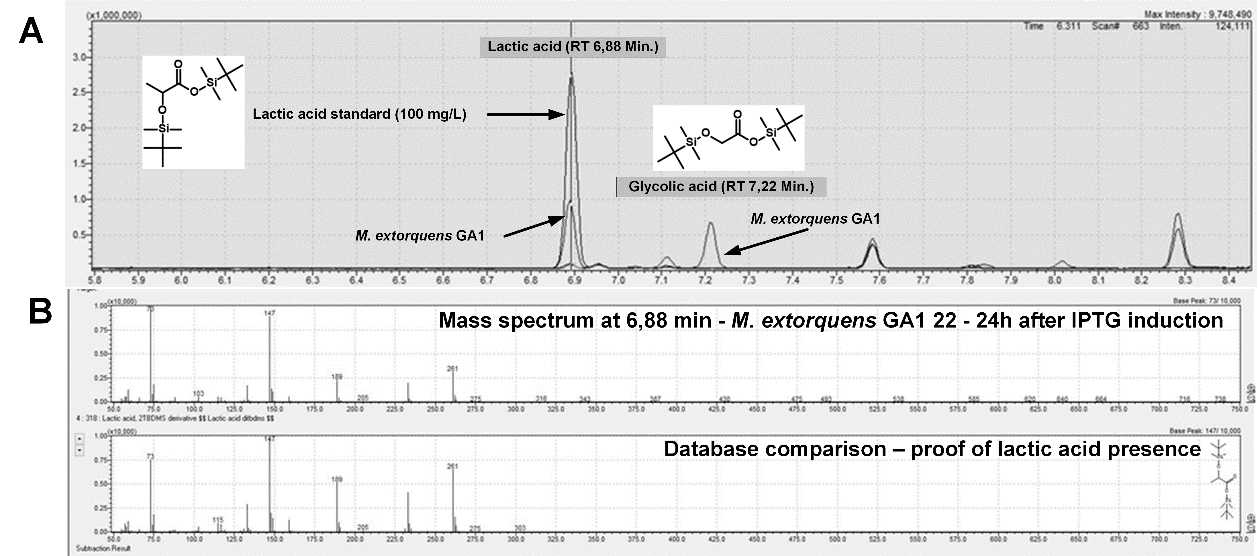


**Figure S6. GC-MS measurements of TBDMS derivatives of a lactic acid standard (100 mg L^-1^) and a sample of M. extorquens GA1 (Mea-GA1) cultivation broth at 22 h after IPTG induction.** The presence of lactic acid was proven by the matching mass spectra of the (**A**) peak at 6.88 min and the (**B**) database (89 % similarity). These measurements were conducted as well for M. extorquens GA2 (Mea-GA2) and GA3 (Mea-GA3), which showed the same prove for lactic acid presence (data not shown).
